# Identification of HIV-1 Reverse Transcriptase-Associated Ribonuclease H Inhibitors Based on 2-Hydroxy-1,4-naphthoquinone Mannich Bases

**DOI:** 10.3390/molecules30030495

**Published:** 2025-01-23

**Authors:** Nhat Quang Tu, Clémence Richetta, Federica Putzu, Olivier Delelis, Khursheed Ahmed, Vijay H. Masand, Rainer Schobert, Enzo Tramontano, Angela Corona, Bernhard Biersack

**Affiliations:** 1 Laboratoire de Biologie et Pharmacologie Appliquée (LBPA), ENS-Paris-Saclay, Centre National de la Recherche Scientifique UMR 8113, Université Paris-Saclay, 91190 Gif-sur-Yvette, France; tunhatquang2@gmail.com (N.Q.T.); clemence.richetta@ens-paris-saclay.fr (C.R.); delelis@lbpa.ens-cachan.fr (O.D.); 2 Department of Life and Environmental Sciences, University of Cagliari Biomedical Section, Laboratory of Molecular Virology, E Block, First Floor, Cittadella Universitaria di Monserrato SS554, 09042 Monserrato, Italy; f.putzu8@studenti.unica.it (F.P.); tramon@unica.it (E.T.); 3 Department of Chemistry, Abeda Inamdar Senior College, University of Pune, Pune 411001, India; khursheed92@rediffmail.com; 4 Department of Chemistry, Vidyabharati Mahavidyalaya, Amravati 444602, India; vijaymasand@gmail.com; 5 Organic Chemistry Laboratory, University of Bayreuth, 95447 Bayreuth, Germany; rainer.schobert@uni-bayreuth.de; 6 Istituto di Ricerca Genetica e Biomedica, Consiglio Nazionale delle Ricerche (CNR), 09042 Monserrato, Italy

**Keywords:** naphthoquinone, lawsone, antiviral drugs, HIV, RNase H, SARS-CoV-2

## Abstract

There is a strong demand for new and efficient antiviral compounds. A series of 2-hydroxy-1,4-naphthoquinone Mannich bases were screened for their HIV-1-RNase H inhibitory activity. An HIV-1-RNase H assay was used to study the RNase H inhibition by the test compounds. Docking of active derivatives into the active site of the enzyme was carried out. Compounds **1e** and **2k** showed distinctly higher HIV-1-RNase H inhibitory activity (IC_50_ = 2.8–3.1 µM) than the known inhibitors RDS1759 and compound **13**. The binding mode and possible interactions of **1e** and **2k** with the HIV-1-RNase H active site were determined using molecular docking, which led to the identification of salient and concealed pharmacophoric features of these molecules. The docking analysis revealed that there are significant differences in the binding mode of these compounds within the active site of the target enzyme. A selection of HIV-1-RNase H-inhibitory Mannich bases was tested for antiviral activity against HIV-1, and compound **2k** showed the highest activity at low toxicity to host cells. The lawsone Mannich bases **1e** and **2k** also underwent a preliminary screening for activity against SARS-CoV-2, and compound **1e** was found to inhibit SARS-CoV-2 replication (IC_50_ = 11.2 µM).

## 1. Introduction

**Figure 1:**
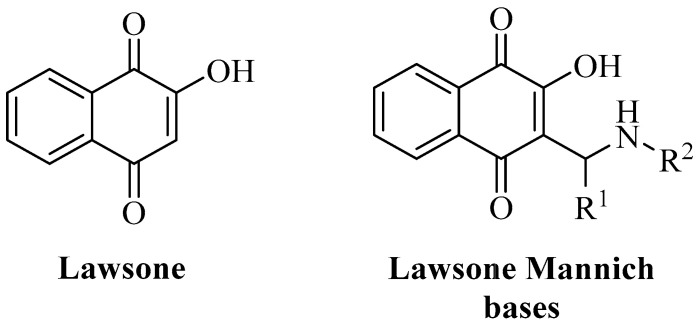
Structures of lawsone (2-hydroxy-1,4-naphthoquinone) and general structure of lawsone Mannich bases (R^1^ = aryl, R^2^ = alkyl/benzyl).

Naphthoquinones are promising starting compounds for the design of new biologically active compounds. 2-Hydroxy-1,4-naphthoquinone (lawsone) is available from henna plants (*Lawsonia inermis*) applied for the treatment of skin diseases in South Asian traditional medicine (Figure 1) [1,2]. A variety of new and potent anticancer, antifungal, and antiparasitic compounds was identified upon chemical modification of lawsone [3,4,5,6,7,8,9,10,11]. In particular, easily prepared lawsone Mannich bases were reported to have anticancer, antiparasitic, and antiviral activities (Figure 1) [12,13,14,15,16]. Due to the currently emerging medical problems associated with viral infections, the antiviral activities of lawsone Mannich bases appear to be of high relevance, warranting further investigations.

For instance, neither cures nor vaccines are available to fight HIV (human immunodeficiency virus) infections to this day. There is no effective approach to eradicate HIV in latently infected tissue reservoirs [17,18]. Infections with HIV (HIV-1 or HIV-2 strains) are wide-spread and ca. 39.9 million infected people (among them ca. 1.4 million children below 15 years) and approximately 630,000 deaths were counted worldwide in 2023 (Available online: www.unaids.org, accessed on 14 September 2024). Acquired immunodeficiency syndrome (AIDS) develops as a consequence of an unhampered infection and has killed about 42.3 million people worldwide since its debut in the 1980s (Available online: www.unaids.org, accessed on 14 September 2024). HIV infections are customarily treated either by highly active antiretroviral therapy (HAART), which mainly includes inhibitors of HIV reverse transcriptase (RT, inhibition of its DNA polymerase subunit RDDP/RNA-dependent DNA polymerase by NRTIs/nucleoside RT inhibitors and NNRTIs/non-nucleoside RT inhibitors), or by inhibitors of HIV integrase (IN) and inhibitors of HIV protease. However, their application is limited due to side-effects and drug resistance [19,20,21]. Co-infection with other infectious diseases such as leishmaniasis or hepatitis B (HBV) poses another eminent threat [22,23].

Recent developments in the field of antiretroviral drugs include second-generation drugs against established HIV targets such as RT (e.g., the NRTIs islatravir and censavudine and the NNRTIs rilpivirine, elsulfavirine, and doravirine) and IN (cabotegravir and dolutegravir). Promising new antiretroviral drug classes are CC5/CCR2 antagonists (e.g., cenicriviroc), as well as inhibitors of attachment, maturation, and capsids (e.g., the capsid inhibitor lenacapavir), which have the potential to overcome resistance to established antivirals [24]. The HIV RT enzyme also harbors an RNase H domain, which has two functions. It unspecifically degrades the RNA strand of the RNA/DNA intermediate during replication and catalyzes the specific hydrolysis of RNA primers during the biosynthesis of pro-viral DNA [25]. The negatively charged DEDD motif and two Mg^2+^ ions form the active site of HIV RNase H required for catalytic hydrolysis of phosphate esters of ribonucleotides [26]. Several inhibitors have already been identified, such as competitive inhibitors of the active site of HIV RNase H and allosteric inhibitors leading to protein destabilization [27]. Moreover, both naïve and drug-treated HIV patients showed a high conservation of the RT-associated RNase H domain, and thus its inhibition could overcome RT resistance and pave the way to new treatment options [28,29]. Considering the critical health care situation in poor countries and the emergence of drug resistance, new affordable drugs for the therapy of viral diseases are needed (Available online: www.dndi.org, accessed on 14 September 2024). In light of current events, the identification of the coronavirus (CoV) ribonucleases nsp14 and nsp15 is of importance and also led to new possible drug candidates for the treatment of CoV infections [30,31].

In the present report, we conducted further investigations in order to identify a conceivable HIV target for lawsone Mannich bases and disclose promising results of some derivatives as inhibitors of the RNase H domain of the HIV-1 RT protein. Computer-aided drug discovery (CADD), particularly molecular docking, has emerged as a method of choice to identify the structural features responsible for binding with the active site residues of the target enzyme. In the present work, we executed flexible docking to capture the easily recognizable and hidden structural features accountable for interacting with the target enzyme. In addition, a docking analysis was performed to understand the significant differences in the binding pose and patterns of newly synthesized molecules inside the active site of the enzyme. Cell-based assays showed that lawsone Mannich bases are able to inhibit HIV-1 replication by reducing the viral transcript amount, hence confirming that HIV-1 RT-associated RNase H functions as a target.

## 2. Results

### 2.1. Lawsone Mannich Bases

**Scheme 1:**
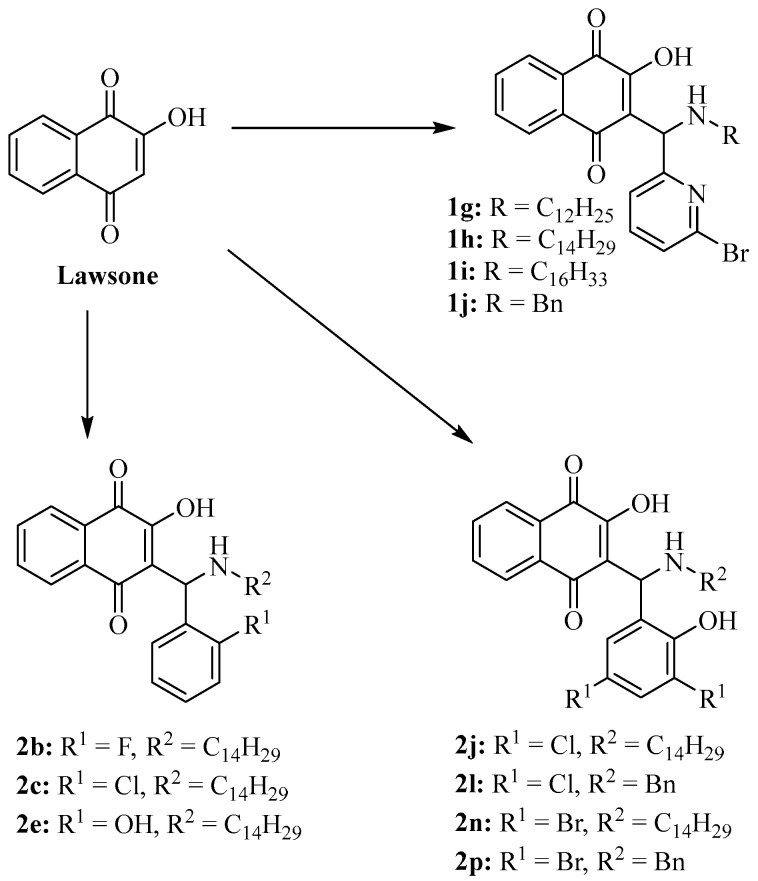
Synthesis of lawsone Mannich bases by Mannich reaction, reagents, and conditions: alkyl/benzyl amine, aryl aldehyde, EtOH, rt, 1 h, 38–74%.

**Figure 2:**
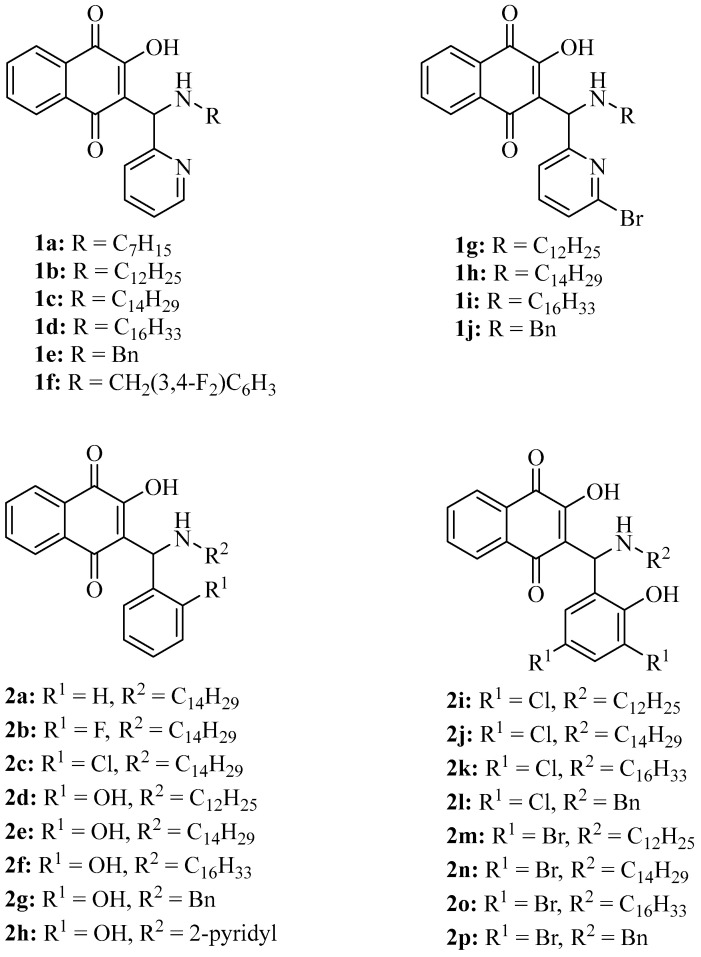
Structures of lawsone Mannich bases used in this study.

The repurposed test compounds **1a**–**f**, **2a**, **2d**, **2f**–**i**, **2k**, **2m**, and **2o** were prepared according to literature procedures (Figure 2) [13,14]. The new lawsone Mannich bases **1g**–**j**, **2b**, **2c**, **2e**, **2j**, **2l**, **2n**, and **2p** were synthesized in the same way by mixing 2-hydroxynaphthoquinone with the corresponding aryl aldehydes and amines in ethanol at room temperature (Scheme 1, Figure 2). These compounds were obtained as yellow-brown or red solids. In total, 26 lawsone Mannich bases were available for the following tests for HIV-1 RNase H inhibition and antiviral activities.

### 2.2. HIV-1 RNase H Inhibition

The compounds were tested for their HIV-1 RT-associated RNase H inhibitory activities (Table 1, Figures S23 and S24). Their activities were compared with the activities of RDS1759 and compound **13** as positive controls, and with lawsone as a negative control [29,32,33]. Compounds **1b** and **1c** of the 2-pyridyl compound series showed strong inhibition of the wild-type HIV1-RNase H (IC_50_ = 4.0–4.4 µM) and were more active than the positive control RDS1759 and in the activity range of our previously published synthetic compound **13** (Table 1). Among the fatty alkyl amino compounds **1a**–**d**, the compounds with the shortest (heptylamino) and the longest (hexadecylamino) linker were less active than the dodecyl- and tetradecylamino derivatives **1b** and **1c**. The benzylamino derivative **1e** was even more active (IC_50_ = 3.1 µM), while fluorination of the benzylamino ring led to lower activity in compound **1f**. The incorporation of a bromine atom at the pyridine ring of compounds **1g**–**j** led to inactive compounds, which stresses the importance of the pyridine ring for RNase H inhibitory activity.

Some lawsone Mannich bases of the salicyl compound series **2a**–**p** also showed promising RNase H inhibitory activities (Table 1). Compounds **2c**, **2e**, **2f**, and **2m** were as active as RDS1759, and **2i** was as active as compound **13**. Compound **2k** was even more active than the positive control compound **13**, and, thus, **2k** is the most active lawsone Mannich base of this study. The 2-chlorophenyl derivative **2c** was slightly more active than its 2-hydroxy congener **2e**, indicating that the hydroxyl group of the salicyl derivatives can be replaced by chlorine. The phenyl compound **2a** and the 2-fluorophenyl derivative **2b** were distinctly less active than the 2-chlorophenyl **2c**. In contrast to the compounds of the 2-pyridyl series **1a–d**, the dodecylamino derivative **2d** was less active than the tetradecyl- and hexadecylamino derivatives **2e** and **2f**. Interestingly, the benzylamino derivative **2g** was inactive, while the bis-pyridine analog **2h** showed moderate activity. As to the 3,5-dihalosalicyl derivatives, the 3,5-dichlorosalicyl compounds were more active than the corresponding 3,5-dibromosalicyl analogs. **2k** was much more active than **2o**, for example. It is also interesting to note that the tetradecylamino derivatives **2j** and **2n** were distinctly less active in this class of 3,5-dihalosalicyl lawsone Mannich bases than the analogous dodecyl- and hexadecylamino compounds **2i**, **2k**, **2m**, and **2o**. Both benzylamino derivatives **2l** and **2p** showed moderate activities and, thus, were more active than the corresponding salicyl derivative **2g**, which lacks the dichloro- and dibromo-substituents of **2l** and **2p**.

### 2.3. HIV-1 RNase H Molecular Docking

**Figure 3:**
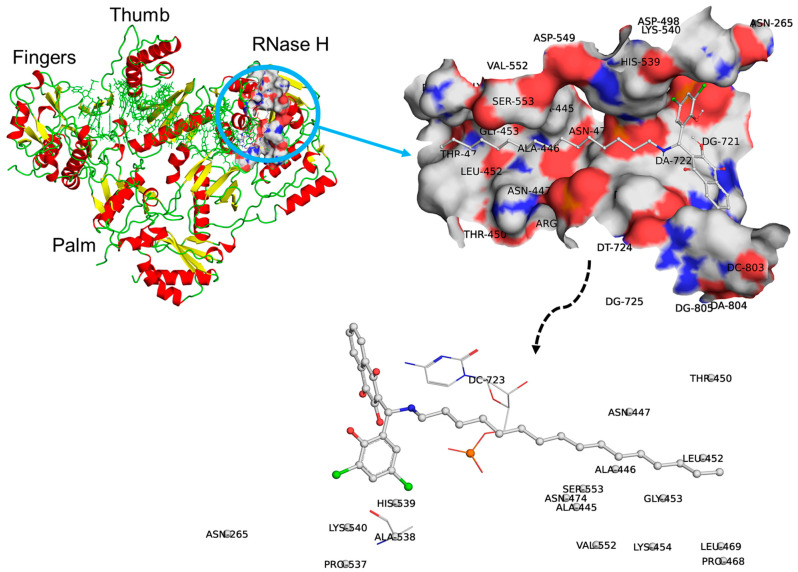
Predicted docking pose of **2k** in the HIV-1-RNase H domain of the PDB structure 6AN2. Different regions of protein (top left) and 3D interactions (top right) along with molecular surface area (colored according to atom type, viz. red: oxygen, blue: nitrogen, and white: carbon).

**Figure 4:**
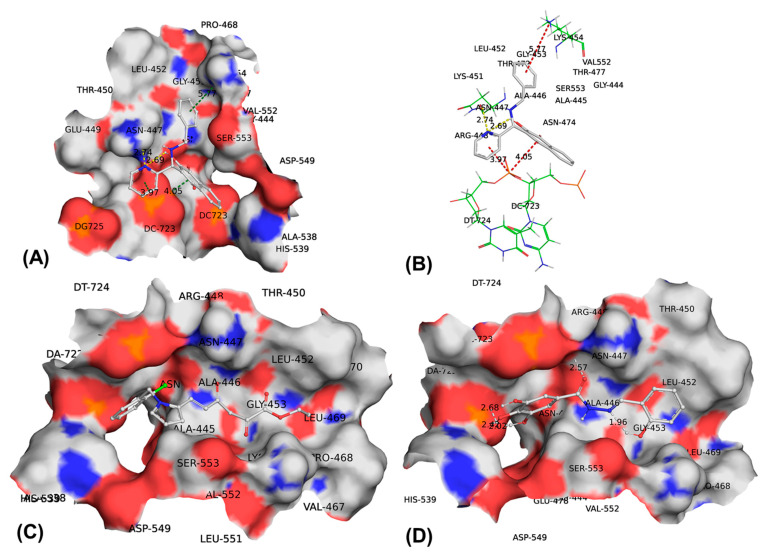
Predicted 3D interaction docking pose of different ligands in the HIV-1-RNase H domain of the PDB structure 6AN2. (**A**) **1e** with surface. (**B**) **1e** without surface. (**C**) RDS1759 with surface and (**D**) compound **13** with surface. Dotted lines indicate polar interactions. Molecular surface area colored according to atom type, viz. red: oxygen, blue: nitrogen, and white: carbon.

Molecular docking was performed in order to rationalize the observed inhibitory efficacies on the HIV-1-RNase H enzymatic activity. Molecular docking was carried out for the best inhibitors **1e** and **2k** as well as compound **13** and RDS1759 to the RNase H domain present in the protein data bank structure 6AN2 (Figure 3 and Figure 4). Docking scores revealed binding energies of −9.035 kcal/mol for compound **2k**, which is in line with its good IC_50_ value against this viral enzyme, and −6.534 kcal/mol for compound **1e**, −6.031 kcal/mol for RDS1749, and −6.107 kcal/mol for compound **13**.

The docking pose for **2k** bound to the active site indicates that the molecule has a variety of interactions with the viral enzyme. It is successful in occupying a major portion of the active site due to the long alkyl chain and the heterocyclic rings. Polar interactions were possible due to the carbonyl groups of the 1,4-dione moiety, while the fatty alkyl chain was responsible for a tight fitting of the molecule inside the active site, forming a ‘Y’ shape with the 1,4-naphthoquinone moiety and the tri-substituted benzene ring acting as a stroke or gore, which are perpendicular to each other, while the fatty alkyl side chain forms a tail (Figure 3). In detail, there are 243 atoms in a total of 17 different amino acids of HIV-RT (ALA445, ALA446, ASN447, THR450, LEU452, GLY453, LYS454, PRO468, LEU469, ASN474, PRO537, ALA538, HIS539, LYS540, VAL552, SER553, and ASN265), with at least one atom within a range of 5 Å of the ligand **2k** (Table S1). The nearest and farthest atoms (within 5 Å) of the protein are HD12 of LEU452 (2.63 Å) and HB2 of HIS539 (4.99 Å), respectively. Likewise, the nearest and farthest atoms (within 5 Å) of the nucleic acid are H42 of base DC723 (2.45 Å) and C4 of base DT724 (4.99 Å), respectively.

**Figure 5:**
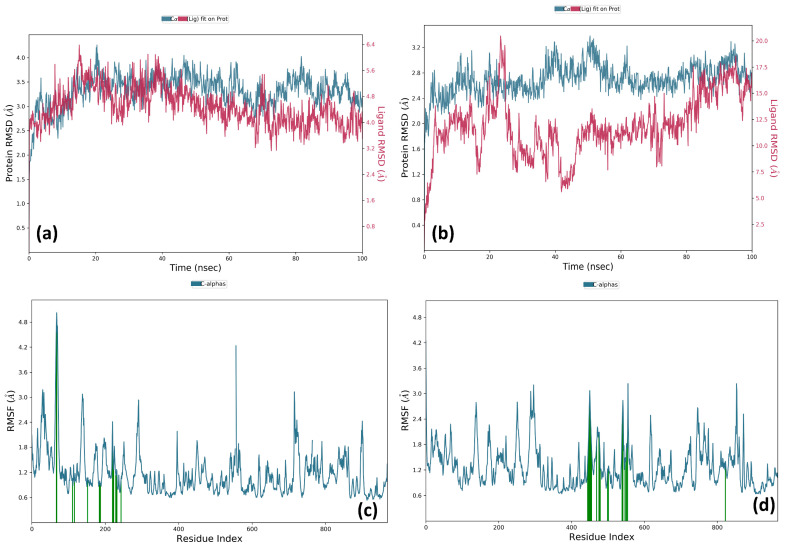
(**a**,**b**) RMSD for holoprotein and complex (6AN2 + **2k**); (**c**,**d**) RMSF for holoprotein and complex (6AN2 + **2k**) (protein residues that interact with the ligand are marked with green vertical bars).

The docking pose for **1e** indicates that the molecule interacts with ALA445, ALA446, ASN447, GLU449, LEU452, GLY453, LYS454, ASN474, HIS539, VAL552, and SER553 (Figure 5a,b). Due to its relatively small size, it is unable to fully occupy the complete active site and adopts a propeller shape with a close proximity to the pyridine ring. The 1,4-naphthoquinone and benzene rings are responsible for hydrophobic interactions, and the pyridine ring for mild polar interactions. More precisely, 181 atoms of 12 amino acids, four DNA bases and four water molecules of HIV-RT have at least one atom within a range of 5 Å of the ligand **1e** (Table S2). The nearest and farthest atoms (within 5 Å) of the protein are NE2 of HIS539 (2.79 Å) and HA of SER553 (4.99 Å), respectively. Likewise, the nearest and farthest atoms (within 5 Å) of the nucleic acid are H5’ of base DC723 (2.18 Å) and H4’ of base DT724 (4.99 Å), respectively. The nitrogen atom of the pyridine rings is accountable for an H-bond with ASN447 at 2.74 Å and an intramolecular H-bond with the -OH group. The carbonyl group of **1e** has a polar interaction with ALA446 with a distance of 3.01 Å.

The docking poses of RDS1759 and compound **13** are presented in Figure 5c,d, respectively. Compound **13** has polar interactions, whereas RDS1759 displays only lipophilic and polar interactions primarily.

### 2.4. Molecular Dynamics Simulations

Molecular dynamics (MD) simulation is widely recognized as a reliable technique for assessing the structural and functional stability of proteins and protein−ligand complexes. MD analysis was carried out to determine the stability and convergence of **2k** bound to HIV-1 reverse transcriptase (RT) in complex with double-stranded DNA (PDB: 6AN2). In the present work, two MD simulations for 100 ns were executed. Each simulation of 100 ns displayed stable conformation while comparing the root mean square deviation (RMSD) values (Figure 5). The RMSD is a metric for calculating the average change in the displacement of a group of atoms in relation to a reference frame. It is calculated for each and every frame of the trajectory.

In order to assess the remaining variance in a 100 ns molecular dynamics (MD) simulation, we analyzed the root mean square fluctuation (RMSF) plot for two specific elements: the holoprotein and the 6AN2-bound most active ligand **2k** (Figure 5c,d). Analysis of the holoprotein’s RMSF revealed notable alterations across various regions, specifically amino acids. The holoprotein RMSD (blue line) stabilized after an initial fluctuation, suggesting that the protein reaches equilibrium around 20–30 ns (Figure 5a). The ligand RMSD (pink line) was relatively stable with minor fluctuations, indicating strong binding stability within the protein pocket (Figure 5a). The **2k** + protein RMSD stabilized after an initial fluctuation but showed slightly higher values compared to holoprotein. The ligand **2k**’s RMSD indicated larger fluctuations, suggesting potential flexibility. Notably, the holo form of the protein displayed greater stability but exhibited higher fluctuations upon ligand **2k** binding. Moreover, the RMSF plots for the potent ligand bound to the 6AN2 protein structure demonstrated a substantial increase in fluctuations within specific amino acid residues, while the remaining residues displayed relatively reduced levels of variance (Figure 5). This could be attributed to the flexible nature of the long carbon chain of ligand **2k**.

**Figure 6:**
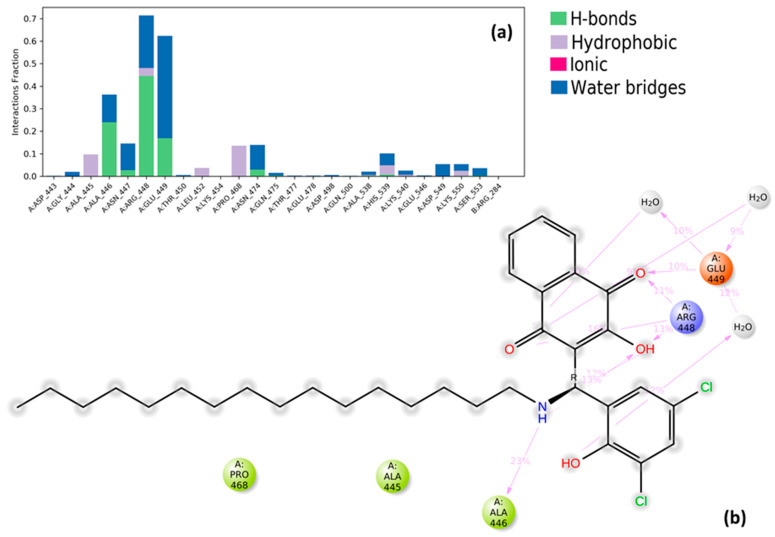
(**a**) Presentation of the 2D interaction of **2k** with the target protein and (**b**) different types of interaction that the ligand formed with the active site residues and solvent during 100 ns of simulation (interaction points with protein residues are highlighted with dotted lines).

From Figure 6a, it can be concluded that the residues ARG448 and GLU449, and the ligand, form strong H-bond interactions. The residue ALA446 also established an H-bond interaction with the ligand through the -NH- fragment. The ligand formed a variety of interactions with the protein (Figure 6b). The residues GLU449 and ARG448 prominently participated in water bridge interactions. Other residues such as ALA445, ALA446, and PRO468 were responsible for close hydrophobic interactions. Thus, H-bonds and lipophilic and mild polar interactions led to a tight binding of **2k** with the protein. The percentages provided near each interaction indicate how often the respective interaction occurred during the simulation, reinforcing the stability of ligand–protein binding.

### 2.5. Inhibition of HIV-1 and SARS-CoV-2 Replication

**Figure 7:**
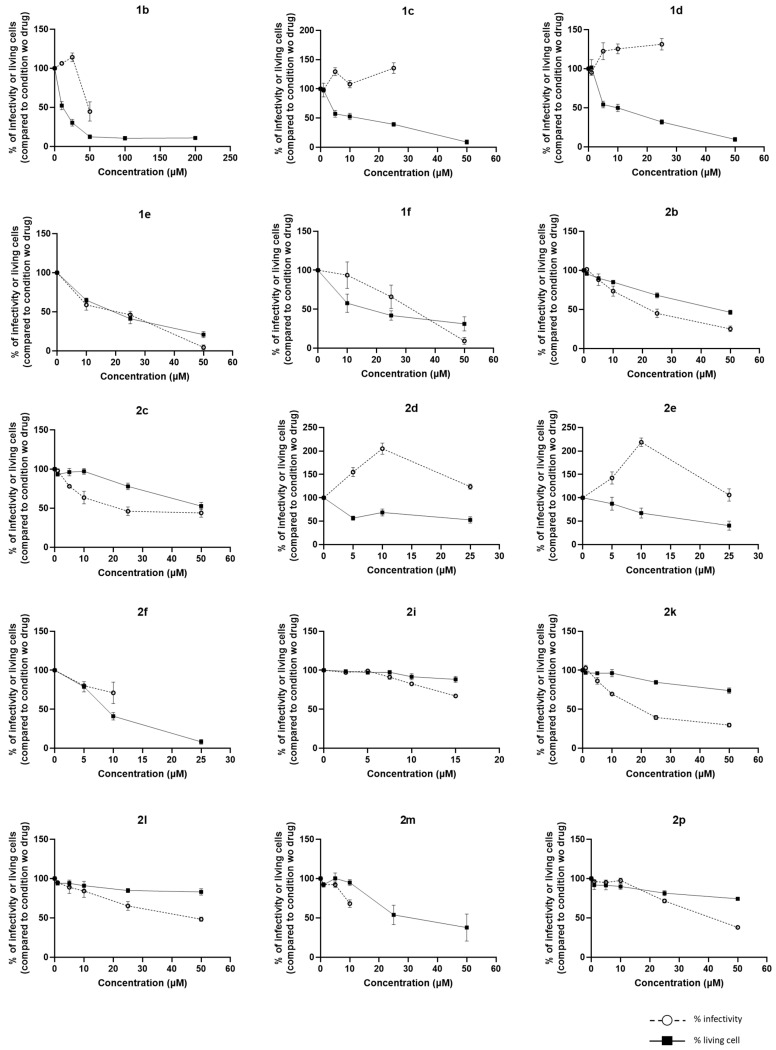
Effect on HIV-1 replication in HeLa P4. Results are expressed as percentage of cellular viability in presence of drug vs. DMSO controls (continuous line) and percentage of HIV-1 infectivity expressed as CPRG reporter activation with respect to DMSO-infected controls (dotted line).

The inhibitory interaction of lawsone Mannich bases with HIV-1 RNase H as an isolated viral molecular target was shown. In order to judge their potential as antiviral drugs, the effects of HIV-1-RNase H inhibitors on HIV-1 replication need to be studied. The compounds that showed an IC_50_ value below 20 µM were tested against HIV-1 replication in cell culture (Figure 7).

In the in vitro study, various concentrations of compounds were initially assessed for toxicity and antiviral efficacy using the HelaP4 cell line. Several compounds displayed cytotoxicity, including the 2-pyridyl derivatives **1b–f** and the salicyl derivatives **2d** and **2f**, while others, such as salicyl **2e** and 3,5-dibromosalicyl **2m**, exhibited no antiviral activity. However, some compounds showed promising antiviral activity. Specifically, 2-fluorophenyl **2b**, 2-chlorophenyl **2c**, the 3,5-dichlorosalicyl derivatives **2i**, **2k**, and **2l**, and the 3,5-dibromosalicyl **2p** effectively inhibited viral infection in HelaP4 cells (Figure 7). Compounds **2b**, **2c**, **2l**, **2p**, **2i,** and **2k** reduced infectivity by 75%, 46%, 48%, 70%, 65%, and 60%, respectively, at their highest non-toxic concentrations.

**Figure 8:**
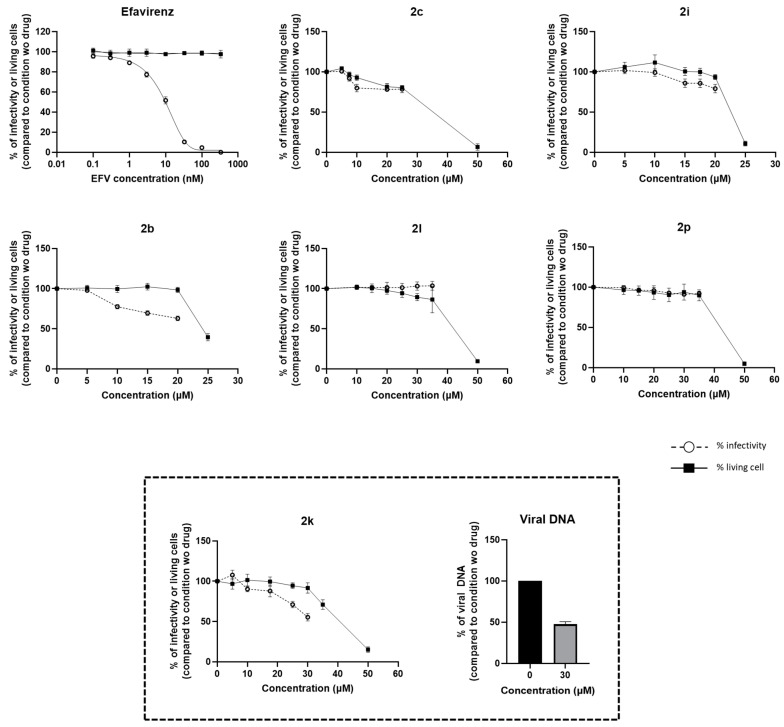
Effect on HIV-1 replication in MT4. Results are expressed as percentage of cellular viability in presence of drug vs. DMSO controls (dotted line) and percentage of HIV-1 infectivity expressed as CPRG reporter activation with respect to DMSO-infected controls (continuous line). Efavirenz was used as positive control.

Subsequently, the six most promising compounds were further tested on the MT4 cell line in order to decipher the viral step targeted by the compounds. The clinically approved NNRTI efavirenz was applied as a positive control. Following the initial colorimetric assays in HelaP4 cells, various concentrations of these compounds were subjected to FACS analysis and the Toxicity ZombieAqua test in MT4 cells (Figure 8). GFP expression was driven by the HIV-1 LTR and indicated the presence of viral DNA. Five compounds (**2b**, **2c**, **2i**, **2l**, and **2p**) displayed either no or weak antiviral effects, as indicated by minimal reductions in fluorescence expression in MT4 cells. Notably, compound **2k**, which had previously inhibited more than 60% of viral infectivity in HelaP4 cells, significantly reduced the percentage of GFP-positive cells by half at a concentration of 30 µM in MT4 cells, without exhibiting any toxicity. Cells treated with this compound were harvested for DNA extraction and subsequent qPCR analysis. The results revealed that 30 µM of **2k** reduced viral DNA levels by over 50%, confirming that the reverse transcription step was targeted by this compound (Figure 8 framed graph).

Compounds showing anti-HIV-1 RT-associated RNase H activity were active against SARS-CoV-2 by targeting nsp14 exoribonuclease [34]. Considering the global COVID-19 pandemic since 2020 and the urgent need for efficient antiviral therapies to treat severe SARS-CoV-2 infections, we briefly investigated the inhibitory effects of the lawsone Mannich bases **1e** and **2k** on SARS-CoV-2 replication in VEROE6-EGFP cells (Table 2). The known 3CLpro inhibitor GC-376 served as a positive control. Since VEROE6 cells express high levels of the ABC transporter P-gp, the non-toxic P-gp inhibitor CP-100356 was added to the tests to prevent drug efflux. While **2k** was inactive here, compound **1e** exhibited moderate inhibitory activity against SARS-CoV-2 replication accompanied by a certain selectivity (selectivity index/SI = 3.15).

### 2.6. ADMET Analysis

**Figure 9:**
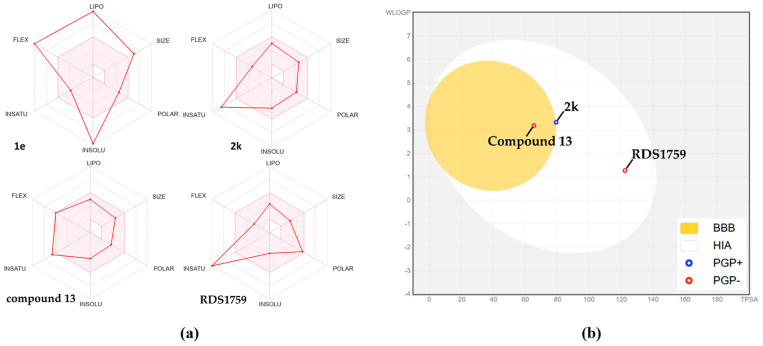
SwissADME results for selected four molecules: (**a**) physico-chemical properties; (**b**) boiled-egg plot.

The SwissADME bioavailability radar charts illustrate six physicochemical parameters, i.e., lipophilicity (LIPO), size, polarity (POLAR), insolubility (INSOL), unsaturation (UNSAT/INSATU), and flexibility (FLEX), for **1e**, **2k**, compound **13**, and RDS1759. The red zone in Figure 9 highlights that the physicochemical properties of these selected compounds are favorable for achieving good in vivo bioavailability.

The SwissADME server also generated a boiled-egg chart that exemplifies human intestinal absorption (white area), blood–brain barrier penetration (yellow area), and the likelihood of **1e**, **2k**, compound **13**, and RDS1759 acting as substrates for PGP+ (blue) or not (red) in relation to permeability glycoprotein (PGP). The position of compound **2k** in the white region indicates an expected adequate oral absorption. Additionally, compound **13** might cause CNS toxicity, as it can cross the blood–brain barrier (represented by the egg yolk area). Compound **1e** has a high WLOGP value (=3.33) and thus falls out of the range of the boiled-egg chart. Based on these results, further modifications seem to be required to optimize the ADMET profiles of compounds **1e** and **2k**.

## 3. Discussion

The present work explored a series of lawsone Mannich bases as HIV-1 RNase H inhibitors. The evaluation of lawsone Mannich bases as HIV-1 RNase H inhibitors led to the identification of the new antiviral drug candidates **1e** and **2k**. Their activities against HIV-1 and SARS-CoV-2 replication are promising and add well to the current knowledge of antiviral lawsone Mannich bases since published data are scarce and limited to inhibitory effects on bovine herpesvirus 5 or herpes simplex virus HSV-1 without detailed mechanistic studies [15,35]. The described lawsone Mannich bases can be prepared by a one-pot multi-component reaction in ethanol at room temperature, which agrees with eco-friendly and cost-effective “green chemistry” principles [36]. Both **1e** and **2k** are hydrophobic compounds and proper formulations might be required for future clinical applications as antiviral drugs. Since the reported anti-herpesvirus lawsone Mannich bases were successfully formulated with liposomes as drug carriers, liposomal systems might be applied for the formulation of **1e** and **2k**, too [37].

The testing of a series of 26 lawsone Mannich bases allows some remarks on structure–activity relationships in terms of RNase H inhibition, HIV-1 replication inhibition, and toxicity to host cells. While certain 2-pyridyl derivatives (**1b**, **1c**, and **1e**) were strong inhibitors of HIV-1 RNase H, bromination of the pyridine ring clearly led to inactivation (**1g**–**j**). In contrast to the strong RNase H inhibitor **1e**, a benzylamino derivative of the 2-pyridyl series, benzylamino compounds were less inhibitory in the salicyl series **2**. However, the benzyl analogs **2l** and **2p** showed antiviral activities. Since heptylamino derivative **1a** was only weakly active against HIV-1 RNase H, longer alkyl chain lengths appear to be required to inhibit this viral enzyme. However, a long alkyl chain alone does not guarantee activity, as can be observed from the activities of some tetradecylamino derivatives (e.g., **2a**, **2j**, and **2n**). Notably, chlorination of the phenyl (**2c**) and salicyl ring systems (**2i** and **2k**) led to promising RNase H inhibition, which might be considered for the design of potent inhibitors in the future. Long fatty alkyl chains, as in the most promising hexadecyl-modified compound **2k**, appear to be more favorable than benzyl substituents in terms of anti-HIV activity. Actually, **2k** combines different chemical features suggested from previous reports, such as a hydrophilic core and long alkyl chains previously applied to naturally derived compounds [38].

Molecular docking studies predict that compounds **2k** and **1e** bind within the HIV-1 RT RNase H domain, interacting with residues belonging to critical functional units of the RNase H domain and found to be conserved among naïve and drug-resistant viral strains [28]. Some of them were also recently reported as critical for the interaction of other RNase H inhibitors such as ASP443 for double-winged galloyl derivates, 3-hydroxypyrimidine-2,4-dione subtypes, and anthraquinones [39,40,41].

Docking calculation revealed a high affinity of **2k**, which is in accordance with its strong inhibitory activity observed in the RNase H inhibition assay. This compound forms a unique ‘Y’ shape within the active site that is stabilized by hydrogen bonds with TRP266 of the p51 subunit and the nucleobase A722, in addition to mild polar interactions at the interface of protein and nucleic acid components of the active site. Compound **1e** displayed a lower affinity for RNase H than **2k**. It interacts with multiple amino acids but does not fully occupy the active site due to its smaller size and relies on hydrophobic and mild polar interactions. These findings highlight the importance of specific structural features for effective RNase H inhibition and offer further structure optimization possibilities. The performed docking study provides a basis for the development of new potent inhibitors targeting the RNase H domain of HIV-1 RT.

The cell-based assays showed that the 2-pyridyl substituent of **1b**–**f** was associated with toxicity to host cells, which is in line with previous reports about the anticancer activity of these compounds against human tumor cells [13]. The 3,5-dichlorosalicyl ring system conveyed antiviral activity but reduced toxicity when compared with the salicyl analogs. This is noteworthy since salicyl-based lawsone Mannich bases were also described as too toxic in tests for bovine herpesvirus activity [15]. Thus, halogenation of the salicyl ring has the potential to improve the selectivity of lawsone Mannich bases. Notably, the chlorinated salicylanilide niclosamide is currently investigated as a COVID-19 drug, and the same compound exhibited activity against HIV-1 [42,43]. Schiff bases with halogenated salicylaldehydes revealed distinct antibacterial and antifungal activities, which underlines the potential of this structural motif for the treatment of multiple infectious diseases [44].

HIV-1 RT-associated RNase H function is the only viral-encoded enzymatic activity that still lacks an approved inhibitor, but is an ever-promising target for drug discovery [27], worth being pursued since HIV-1 also exhibits the ability to overcome the last generation of integrase inhibitors with unprecedentedly described adaptive mechanisms [45,46]. The RT-associated RNase H domain was found to be highly conserved in HIV patients that have received RT inhibitor therapy [28,29]. Thus, RNase H inhibitors might be clinically applied as second-line therapy in case of emerging RT resistance or in combination with other classes of approved inhibitors to prevent RT resistance selection in the HIV patient. However, since mutations of the RNase H domain were reported, it cannot be excluded that treatment with RNase H-targeting drugs can lead to inhibitor-insensitive RNase H domains [47]. A combination therapy might limit the selection of such RNase H mutations and possible resistance to RNase H inhibitors. Lawsone Mannich base **2k**, which was the most active compound against HIV-1 RNase H, also inhibited HIV-1 replication and reduced viral DNA levels in infected cells. Thus, a correlation between RNase H interference and suppressed virus replication can be assumed for **2k**.

## 4. Materials and Methods

### 4.1. Chemistry

Starting compounds were purchased from Sigma-Aldrich (Darmstadt, Germany), Alfa Aesar (Karlsruhe, Germany), and TCI (Zwijndrecht, Belgium). Solvents were used without further purification. The known compounds **1a**–**f**, **2a**, **2d**, **2f**–**i**, **2k**, **2m**, and **2o** were prepared according to literature procedures [13,14].

#### 4.1.1. Synthesis of New Compounds

##### 3-[(Dodecylamino)(6-bromo-2-pyridyl)methyl]-2-hydroxy-1,4-naphthoquinone (**1g**)—Typical Procedure

2-Hydroxy-1,4-naphthoquinone (218 mg, 1.25 mmol) was suspended in EtOH (15 mL), dodecylamine (254 mg, 1.37 mmol) was added, and then the resulting solution was stirred at room temperature for 5 min. 6-Bromo-2-pyridinecarboxaldehyde (279 mg, 1.5 mmol) was added and the reaction mixture was stirred at room temperature for 1 h. The formed precipitate was collected, washed with EtOH, and then dried in a vacuum. Yield: 425 mg (0.81 mmol, 65%); orange-red solid of mp 149–150 °C; *υ*_max_(ATR)/cm^−1^ 3151, 2921, 2852, 2614, 1682, 1583, 1551, 1505, 1471, 1438, 1424, 1407, 1382, 1273, 1232, 1158, 1122, 1086, 1062, 987, 902, 856, 824, 793, 747, 733, 706, 678, 666; ^1^H NMR (300 MHz, CDCl_3_) *δ* 0.85 (3 H, t, *J* = 6.7 Hz), 1.1–1.4 (16 H, m), 1.4–1.5 (2 H, m), 1.9–2.0 (2 H, m), 3.1–3.2 (1 H, m), 3.3–3.4 (1 H, m), 5.71 (1 H, s), 7.2–7.5 (5 H, m), 7.7–7.8 (2 H, m), 9.2–9.4 (2 H, m); ^13^C NMR (75.5 MHz, CDCl_3_) *δ* 14.1, 22.7, 26.6, 26.9, 29.1, 29.3, 29.4, 29.5, 29.6, 31.9, 47.5, 58.4, 111.2, 121.2, 125.4, 126.2, 127.1, 131.0, 131.3, 133.7, 134.1, 139.7, 140.2, 157.1, 170.7, 181.0, 184.8; *m*/*z* (%) 431 (2), 344 (11), 342 (12), 260 (23), 258 (22), 192 (21), 105 (22), 91 (100), 77 (45), 65 (41), 51 (32); anal. calcd. for C_28_H_35_BrN_2_O_3_: C, 63.76; H, 6.69. Found: C, 63.90; H, 6.64.

##### 3-[(Tetradecylamino)(6-bromo-2-pyridyl)methyl]-2-hydroxy-1,4-naphthoquinone (**1h**)

Analogously to the synthesis of **1g**, compound **1h** was obtained from 2-hydroxy-1,4-naphthoquinone (218 mg, 1.25 mmol), tetradecylamine (293 mg, 1.37 mmol), and 6-bromo-2-pyridinecarboxaldehyde (279 mg, 1.5 mmol). Yield: 290 mg (0.52 mmol, 42%); orange-red solid of mp 150–151 °C; *υ*_max_(ATR)/cm^−1^ 3090, 2922, 2852, 1686, 1585, 1553, 1528, 1506, 1466, 1438, 1423, 1403, 1377, 1316, 1267, 1233, 1187, 1152, 1122, 1029, 988, 919, 894, 874, 858, 824, 790, 741, 731, 706, 680, 663; ^1^H NMR (300 MHz, CDCl_3_) *δ* 0.86 (3 H, t, *J* = 6.7 Hz), 1.1–1.4 (20 H, m), 1.4–1.5 (2 H, m), 1.9–2.0 (2 H, m), 3.1–3.2 (1 H, m), 3.3–3.4 (1 H, m), 5.70 (1 H, s), 7.2–7.5 (5 H, m), 7.7–7.8 (2 H, m), 9.3–9.4 (2 H, m); ^13^C NMR (75.5 MHz, CDCl_3_) *δ* 14.1, 22.7, 26.6, 26.8, 29.1, 29.3, 29.4, 29.5, 29.6, 29.7, 31.9, 47.3, 58.3, 111.2, 121.1, 125.4, 126.2, 126.9, 131.0, 131.3, 133.7, 134.2, 139.5, 140.1, 157.4, 170.7, 181.0, 184.8; HR-MS (ESI, *m*/*z*) for C_30_H_40_O_3_N_2_^81^Br [M^+^] calcd. 557.21964, found 557.21817.

##### 3-[(Hexadecylamino)(6-bromo-2-pyridyl)methyl]-2-hydroxy-1,4-naphthoquinone (**1i**)

Analogously to the synthesis of **1g**, compound **1i** was obtained from 2-hydroxy-1,4-naphthoquinone (218 mg, 1.25 mmol), hexadecylamine (330 mg, 1.37 mmol), and 6-bromo-2-pyridinecarboxaldehyde (279 mg, 1.5 mmol). Yield: 560 mg (0.96 mmol, 77%); orange-red solid of mp 130 °C; *υ*_max_(ATR)/cm^−1^ 3074, 2921, 2849, 1685, 1586, 1554, 1528, 1505, 1468, 1439, 1421, 1403, 1378, 1268, 1232, 1188, 1156, 1123, 1084, 1026, 988, 920, 894, 881, 856, 824, 790, 741, 730, 706, 681, 664; ^1^H NMR (300 MHz, CDCl_3_) *δ* 0.86 (3 H, t, *J* = 6.7 Hz), 1.1–1.4 (24 H, m), 1.4–1.5 (2 H, m), 1.9–2.0 (2 H, m), 3.1–3.2 (1 H, m), 3.3–3.4 (1 H, m), 5.70 (1 H, s), 7.2–7.5 (5 H, m), 7.8–7.9 (2 H, m), 9.3–9.6 (2 H, m); ^13^C NMR (75.5 MHz, CDCl_3_) *δ* 14.1, 22.7, 26.6, 26.8, 29.1, 29.3, 29.4, 29.5, 29.6, 29.7, 31.9, 47.4, 58.3, 111.2, 121.1, 125.4, 126.2, 127.0, 131.0, 131.3, 133.7, 134.2, 139.5, 140.1, 157.3, 170.7, 181.0, 184.8; HR-MS (ESI, *m*/*z*) for C_32_H_44_O_3_N_2_^81^Br [M^+^] calcd. 585.25094, found 585.24903.

##### 3-[(Benzylamino)(6-bromo-2-pyridyl)methyl]-2-hydroxy-1,4-naphthoquinone (**1j**)

Analogously to the synthesis of **1g**, compound **1j** was obtained from 2-hydroxy-1,4-naphthoquinone (218 mg, 1.25 mmol), benzylamine (148 mg, 1.37 mmol), and 6-bromo-2-pyridinecarboxaldehyde (279 mg, 1.5 mmol). Yield: 310 mg (0.69 mmol, 55%); red solid of mp 168–169 °C; *υ*_max_(ATR)/cm^−1^ 3065, 3034, 2940, 2771, 2721, 2596, 1679, 1583, 1538, 1499, 1422, 1387, 1303, 1287, 1270, 1236, 1158, 1121, 1089, 1053, 1034, 1009, 988, 940, 911, 895, 830, 793, 745, 721, 706, 699, 681, 665; ^1^H NMR (300 MHz, CDCl_3_) *δ* 4.05 (1 H, d, *J* = 12.8 Hz), 4.20 (1 H, d, *J* = 12.8 Hz), 5.76 (1 H, s), 7.2–7.6 (10 H, m), 7.8–8.0 (2 H, m), 9.7–9.8 (2 H, m); ^13^C NMR (75.5 MHz, CDCl_3_) *δ* 48.8, 57.2, 108.7, 120.6, 125.0, 125.4, 126.5, 128.3, 128.6, 129.7, 130.4, 131.4, 133.1, 134.2, 139.3, 157.9, 171.2, 179.4, 183.7; *m*/*z* (%) 297 (12), 295 (12), 199 (97), 197 (100), 172 (16), 105 (21), 43 (34); anal. calcd. for C_23_H_17_BrN_2_O_3_: C, 61.49; H, 3.81. Found: C, 61.58; H, 3.83.

##### 3-[(Tetradecylamino)(2-fluorophenyl)methyl]-2-hydroxy-1,4-naphthoquinone (**2b**)

Analogously to the synthesis of **1g**, compound **2b** was obtained from 2-hydroxy-1,4-naphthoquinone (218 mg, 1.25 mmol), tetradecylamine (293 mg, 1.37 mmol), and 2-fluorobenzaldehyde (158 µL, 1.5 mmol). Yield: 270 mg (0.55 mmol, 44%); orange solid of mp 139–140 °C; *υ*_max_(ATR)/cm^−1^ 2918, 2851, 1672, 1617, 1590, 1530, 1492, 1468, 1364, 1314, 1262, 1234, 1164, 1099, 1038, 958, 892, 842, 824, 803, 787, 771, 758, 732, 720, 693, 664; ^1^H NMR (300 MHz, CDCl_3_) *δ* 0.85 (3 H, t, *J* = 6.7 Hz), 1.1–1.4 (20 H, m), 1.4–1.5 (2 H, m), 1.8–1.9 (2 H, m), 2.9–3.1 (2 H, m), 5.97 (1 H, s), 7.2–7.5 (5 H, m), 6.9–7.0 (2 H, m), 7.1–7.2 (1 H, m), 7.3–7.5 (2 H, m), 7.6–7.9 (3 H, m), 9.5–9.7 (1 H, br s), 10.2–10.3 (1 H, br s); ^13^C NMR (75.5 MHz, CDCl_3_) *δ* 14.1, 22.7, 26.6, 26.7, 29.1, 29.3, 29.4, 29.5, 29.6, 31.9, 46.1, 53.2, 112.0, 115.3–115.6 (m), 124.0–124.2 (m), 124.6, 125.5, 126.1, 130.3, 131.1, 133.8–134.0 (m), 158.6–161.9 (m), 170.7, 181.3, 184.8; HR-MS (ESI, *m*/*z*) for C_31_H_41_O_3_NF [M + H^+^] calcd. 494.30650, found 494.30520.

##### 3-[(Tetradecylamino)(2-chlorophenyl)methyl]-2-hydroxy-1,4-naphthoquinone (**2c**)

Analogously to the synthesis of **1g**, compound **2c** was obtained from 2-hydroxy-1,4-naphthoquinone (218 mg, 1.25 mmol), tetradecylamine (293 mg, 1.37 mmol), and 2-chlorobenzaldehyde (169 µL, 1.5 mmol). Yield: 240 mg (0.47 mmol, 38%); orange solid of mp 146–147 °C; *υ*_max_(ATR)/cm^−1^ 2919, 2851, 1679, 1618, 1590, 1526, 1470, 1440, 1362, 1322, 1269, 1224, 1162, 1039, 971, 872, 792, 771, 759, 732, 684, 663; ^1^H NMR (300 MHz, CDCl_3_) *δ* 0.85 (3 H, t, *J* = 6.7 Hz), 1.1–1.4 (20 H, m), 1.3–1.4 (2 H, m), 1.8–1.9 (2 H, m), 2.8–2.9 (1 H, m), 3.1–3.2 (1 H, m), 6.10 (1 H, s), 7.1–7.2 (2 H, m), 7.3–7.5 (3 H, m), 7.6–7.8 (3 H, m), 8.3–8.5 (1 H, br s), 10.9–11.1 (1 H, br s); ^13^C NMR (75.5 MHz, CDCl_3_) *δ* 14.1, 22.7, 26.7, 26.9, 29.1, 29.3, 29.4, 29.5, 29.6, 32.0, 46.0, 56.9, 112.7, 125.5, 126.1, 127.6, 129.8, 130.1, 130.4, 131.1, 133.8, 133.9, 134.5, 134.6, 170.1, 181.4, 184.8; HR-MS (ESI, *m*/*z*) for C_31_H_41_O_3_NCl [M + H^+^] calcd. 510.27695, found 510.27572.

##### 3-[(Tetradecylamino)(2-hydroxyphenyl)methyl]-2-hydroxy-1,4-naphthoquinone (**2e**)

Analogously to the synthesis of **1g**, compound **2e** was obtained from 2-hydroxy-1,4-naphthoquinone (218 mg, 1.25 mmol), tetradecylamine (293 mg, 1.37 mmol), and salicylaldehyde (313 µL, 3.0 mmol). Yield: 565 mg (1.15 mmol, 46%); red solid of mp 144–146 °C; *υ*_max_(ATR)/cm^−1^ 3216, 3072, 2921, 2851, 2743, 2603, 1679, 1590, 1514, 1455, 1366, 1275, 1236, 1156, 1099, 1041, 972, 898, 849, 829, 793, 758, 738, 721, 695, 665; ^1^H NMR (300 MHz, CDCl_3_) *δ* 0.85 (3 H, t, *J* = 6.7 Hz), 1.1–1.4 (20 H, m), 1.4–1.5 (2 H, m), 1.8–1.9 (2 H, m), 3.0–3.2 (2 H, m), 5.84 (1 H, s), 6.8–6.9 (2 H, m), 7.0–7.1 (1 H, m), 7.3–7.6 (3 H, m), 7.7–7.8 (1 H, m); ^13^C NMR (75.5 MHz, CDCl_3_) *δ* 14.1, 22.7, 26.6, 29.0, 29.3, 29.4, 29.5, 29.6, 29.7, 31.9, 46.7, 55.4, 112.8, 118.4, 120.3, 123.1, 125.6, 126.2, 127.1, 129.5, 130.9, 131.5, 133.9, 134.0, 154.8, 170.1, 183.0, 184.5; *m*/*z* (%) 278 (72), 249 (20), 221 (100), 194 (22), 165 (54), 105 (52), 77 (36), 55 (33), 44 (55); anal. calcd. for C_31_H_41_NO_4_: C, 75.73; H, 8.41. Found: C, 75.67; H, 8.44.

##### 3-[(Tetradecylamino)(3,5-dichloro-2-hydroxyphenyl)methyl]-2-hydroxy-1,4-naphthoquinone (**2j**)

Analogously to the synthesis of **1g**, compound **2j** was obtained from 2-hydroxy-1,4-naphthoquinone (218 mg, 1.25 mmol), tetradecylamine (293 mg, 1.37 mmol), and 3,5-dichlorosalicylaldehyde (286 mg, 1.5 mmol). Yield: 291 mg (0.52 mmol, 42%); orange-red solid of mp 147–149 °C; *υ*_max_(ATR)/cm^−1^ 3198, 3069, 2922, 2852, 2540, 1680, 1589, 1505, 1468, 1375, 1273, 1230, 1169, 1156, 1095, 1055, 990, 942, 864, 824, 796, 756, 734, 718, 699, 666; ^1^H NMR (300 MHz, CDCl_3_) *δ* 0.85 (3 H, t, *J* = 6.7 Hz), 1.1–1.4 (20 H, m), 1.3–1.5 (2 H, m), 1.7–1.9 (2 H, m), 3.0–3.2 (2 H, m), 5.8–5.9 (1 H, br s), 7.0–7.1 (1 H, m), 7.3–7.4 (2 H, m), 7.5–7.7 (2 H, m), 7.9–8.0 (1 H, m); ^13^C NMR (75.5 MHz, CDCl_3_) *δ* 14.1, 22.7, 26.4, 27.0, 29.0, 29.3, 29.4, 29.5, 29.6, 29.7, 30.5, 31.9, 47.2, 55.4, 57.8, 112.1, 124.7, 125.9, 126.5, 128.3, 129.5, 130.7, 131.9, 132.5, 133.7, 134.4, 135.2, 149.8, 163.1, 170.1, 183.2, 184.5; *m*/*z* (%) 387 (35), 385 (54), 244 (100), 203 (57), 175 (48), 105 (45), 41 (55); anal. calcd. for C_31_H_39_Cl_2_NO_4_: C, 66.42; H, 7.01. Found: C, 66.55; H, 6.98.

##### 3-[(Benzylamino)(3,5-dichloro-2-hydroxyphenyl)methyl]-2-hydroxy-1,4-naphthoquinone (**2l**)

Analogously to the synthesis of **1g**, compound **2l** was obtained from 2-hydroxy-1,4-naphthoquinone (218 mg, 1.25 mmol), benzylamine (295 mg, 2.75 mmol), and 3,5-dichlorosalicylaldehyde (573 mg, 3.0 mmol). Yield: 556 mg (1.22 mmol, 49%); red solid of mp 149–150 °C; *υ*_max_(ATR)/cm^−1^ 3069, 3034, 2960, 2719, 2526, 1685, 1591, 1515, 1471, 1459, 1419, 1373, 1272, 1237, 1216, 1167, 1157, 1092, 1066, 1036, 997, 955, 915, 857, 824, 796, 757, 734, 721, 696, 659; ^1^H NMR (300 MHz, DMSO-d_6_) *δ* 4.16 (2 H, s), 5.60 (1 H, s), 7.3–7.4 (6 H, m), 7.6–7.7 (2 H, m), 7.7–7.8 (1 H, m), 7.8–7.9 (1 H, m), 7.9–8.0 (1 H, m); ^13^C NMR (75.5 MHz, DMSO-d_6_) *δ* 42.3, 49.4, 52.9, 58.4, 110.4, 122.3, 122.5, 125.1, 125.7, 125.9, 127.7, 127.9, 128.2, 128.5, 128.7, 128.8, 130.3, 131.3, 131.9, 132.6, 133.7, 134.0, 149.6, 165.7, 171.4, 180.2, 183.3; *m*/*z* (%) 347 (4), 345 (6), 280 (22), 278 (31), 91 (100), 65 (45); anal. calcd. for C_24_H_17_Cl_2_NO_4_: C, 63.45; H, 3.77. Found: C, 63.53; H, 3.80.

##### 3-[(Tetradecylamino)(3,5-dibromo-2-hydroxyphenyl)methyl]-2-hydroxy-1,4-naphthoquinone (**2n**)

Analogously to the synthesis of **1g**, compound **2n** was obtained from 2-hydroxy-1,4-naphthoquinone (218 mg, 1.25 mmol), tetradecylamine (293 mg, 1.37 mmol), and 3,5-dibromosalicylaldehyde (420 mg, 1.5 mmol). Yield: 468 mg (0.72 mmol, 58%); orange-red solid of mp 150–151 °C; *υ*_max_(ATR)/cm^−1^ 3043, 2921, 2852, 1676, 1589, 1505, 1466, 1434, 1367, 1303, 1272, 1234, 1149, 1093, 1054, 992, 917, 864, 836, 823, 796, 748, 736, 713, 690, 667, 655; ^1^H NMR (300 MHz, CDCl_3_) *δ* 0.85 (3 H, t, *J* = 6.7 Hz), 1.1–1.4 (20 H, m), 1.4–1.5 (2 H, m), 1.8–1.9 (2 H, m), 3.0–3.2 (2 H, m), 5.83 (1 H, s), 7.5–7.7 (5 H, m), 7.8–7.9 (1 H, m); ^13^C NMR (75.5 MHz, CDCl_3_) *δ* 14.1, 22.7, 26.4, 26.7, 29.0, 29.4, 29.5, 29.6, 29.7, 31.9, 47.3, 55.4, 111.9, 113.8, 125.9, 126.6, 129.3, 130.7, 131.9, 133.7, 134.4, 135.0, 151.2, 170.1, 183.3, 184.5; HR-MS (ESI, *m*/*z*) for C_31_H_40_O_4_NBr^81^Br [M + H^+^] calcd. 650.12981, found 650.12777.

##### 3-[(Benzylamino)(3,5-dibromo-2-hydroxyphenyl)methyl]-2-hydroxy-1,4-naphthoquinone (**2p**)

Analogously to the synthesis of **1g**, compound **2p** was obtained from 2-hydroxy-1,4-naphthoquinone (218 mg, 1.25 mmol), benzylamine (295 mg, 2.75 mmol), and 3,5-dibromosalicylaldehyde (840 mg, 3.0 mmol). Yield: 780 mg (1.44 mmol, 58%); red solid of mp 158 °C; *υ*_max_(ATR)/cm^−1^ 3062, 3031, 2964, 2726, 2538, 1685, 1591, 1512, 1471, 1415, 1372, 1270, 1236, 1147, 1091, 1065, 1032, 994, 950, 908, 855, 823, 748, 734, 721, 689, 658; ^1^H NMR (300 MHz, DMSO-d_6_) *δ* 4.15 (2 H, s), 5.57 (1 H, s), 7.3–7.4 (5 H, m), 7.6–7.7 (2 H, m), 7.7–7.8 (1 H, m), 7.8–7.9 (1 H, m), 7.9–8.0 (1 H, m); ^13^C NMR (75.5 MHz, DMSO-d_6_) *δ* 42.3, 49.4, 52.9, 58.0, 105.5, 110.2, 110.6, 112.5, 118.2, 121.9, 125.1, 125.7, 127.7, 127.9, 128.4, 128.5, 128.6, 128.8, 129.1, 130.4, 131.2, 131.9, 133.2, 133.7, 134.0, 137.0, 137.9, 151.1, 151.5, 162.6, 165.6, 171.4, 180.3, 183.2; *m*/*z* (%) 437 (3), 435 (6), 369 (42), 106 (36), 91 (100), 65 (68), 51 (37); anal. calcd. for C_24_H_17_Br_2_NO_4_: C, 53.07; H, 3.16. Found: C, 53.14; H, 3.11.

### 4.2. HIV1-RDDP-Independent-RNase H Inhibition

HIV-1 RT-associated RNase H inhibition assays were carried out in a 384 multiwell format, in 30 µL reaction volume containing 50 mM Tris–HCl buffer pH 7.8, 6 mM MgCl_2_, 1 mM dithiothreitol (DTT), 80 mM KCl, 0.25 µM hybrid RNA/DNA 5’-GAUCUGAGCCUGGGAGCU-Fluorescin-3’ 5’-Dabcyl-AGCTCCCAGGCTCAGATC-3’ increasing concentrations of inhibitor, whose dilutions were made in DMSO, and 50 nM of HIV-1 RT. The reaction mixture was incubated for 15 min at 37 °C and products were measured with a multilabel counter plate reader VictorNivo5 (PerkinElmer, Waltham, MA, USA) equipped with filters for 490/528 nm. IC_50_, the concentration able to inhibit 50% of enzymatic activity, was calculated by non-linear regression curve fit using Prisme v9.0. All experiments were conducted in triplicate.

### 4.3. Molecular Modeling

For molecular docking analysis, the 3D structure of the target enzyme HIV-RT was retrieved from a protein data bank (Available online: www.rcsb.org, accessed on 4 December 2024). The structure PDB-6AN2 was selected on the basis of X-ray resolution (2.70 Å) and completion of the sequence for the p66 subunit, which was used for molecular docking. During protein preparation, we removed subunit p51, another heterodimer, and retained water molecules, as water not only stabilizes ligand interactions but also plays a crucial biological role in determining specificity. Before conducting the docking study, the co-crystallized ligand (D4T) was removed. The compounds were docked into the active site where the co-crystallized ligand was originally bound. The protein preparation process included the adjustment of non-standard residues, the addition of hydrogens, and the assignment of partial charges (Kollman charges). Before actual molecular docking, Ramachandran’s plot was used to check the health of the protein, which confirmed that the protein was suitable for molecular docking (Figure 3). All compounds were docked in the active site of the target enzyme. Flexible docking was accomplished using NRGSuite, which is a free plugin used along with PyMOL (Available online: www.pymol.org, accessed on 4 December 2024). The software detects target binding sites for docking simulations using surface cavities and executes the molecular docking using a genetic algorithm for conformational search. The standard procedure mentioned in the manual of the software was followed to obtain optimum performance and better results. For flexible–flexible docking, default settings were used along with the following changes: binding site input method—spherical shape (diameter: 18 Å); spacing of three dimensional grid—0.375 Å; side chain flexibility—yes; ligand flexibility—yes; ligand pose as reference—no; constraints—no; Hetero groups—included water molecules; van der Walls permeability—0.1; solvent types—no type; number of chromosomes—1000; number of generations—10,000; fitness model—share; reproduction model—population boom; and number of top complexes—5. Further details are available in the Supplementary Materials.

### 4.4. Molecular Dynamics Simulations and MM-GBSA Analysis

MD simulations were conducted using Desmond 2023-4 [48] and focused on exploring the interactions between the ligand **2k** and the target protein. For this reason, the holo form of HIV-1 reverse transcriptase (RT) in complex with double-strand DNA was also studied. The primary aim was to investigate the binding behavior and dynamic properties of **2k** in complex with the protein. Consistent experimental protocols were maintained across all trials and data acquisition steps to ensure reproducibility [49].

Simulations utilized the OPLS-2005 (Schrödinger, New York, NY, USA) force field and an explicit solvent environment comprising simple point charge (SPC) water molecules [50]. After solvating the system, energy minimization was performed using the OPLS-2005 force field followed by a stepwise relaxation process [51]. The initial system configuration included a cubic simulation box with dimensions of 7.0 × 7.0 × 7.0 nm, as defined in Desmond. Sodium ions (Na^+^) were introduced to neutralize the system, while a 0.15 M sodium chloride (NaCl) solution was added to replicate physiological ionic conditions.

An equilibration phase, lasting for 50 nanoseconds, was carried out under an NVT ensemble to stabilize the system, followed by an additional 12-nanosecond equilibration and minimization phase under an NPT ensemble. The combination of 50 nanoseconds of NVT equilibration and 12 nanoseconds of NPT equilibration ensures that the system is both thermodynamically and structurally stable. This preparation is critical before running the production phase of molecular dynamics simulations, as it prevents non-physical artifacts and ensures realistic modeling of biomolecular interactions. Temperature control at 37 °C was achieved using a Nosé–Hoover chain thermostat, and pressure stabilization at 1 bar was maintained with a Martyna–Tuckerman–Klein barostat, which featured a relaxation period of 2 ps [52,53]. Long-range electrostatic interactions were calculated using the particle mesh Ewald (PME) method with a cutoff distance of 9 Å for Coulomb interactions. Time-step integration employed a RESPA scheme with a step size of 2 femtoseconds [52,53,54,55].

Stability and interaction dynamics were evaluated through key metrics such as root mean square deviation (RMSD), root mean square fluctuation (RMSF), and hydrogen bond (H-bond) analysis. These metrics provided valuable insights into the structural stability and interaction mechanisms of the ligand–protein complexes under physiological conditions. Further details are available in the Supplementary Materials.

### 4.5. Inhibition of HIV-1 Replication

#### 4.5.1. Cells

HeLa-P4 and MT4 cells were cultured in Dulbecco’s Modified Eagle Medium (DMEM) and Roswell Park Memorial Institute (RPMI) 1640, respectively (Gibco, Waltham, MA, USA), supplemented with 10% fetal bovine serum (Eurobio scientific #CVFSVF00-01) and 1% penicillin/streptomycin (100 units/mL) (Gibco). All cell lines were incubated at 37 °C, 5% CO_2_.

#### 4.5.2. Virus

This study utilized the pnl4.3-GFP delta env viral strain, which has the fluorescent protein reporter EGFP inserted between the envelope and nef open reading frames. HIV-1 virus stocks were produced by transfecting 293T cells using the Calcium Phosphate method. Envelope-defective viruses were pseudotyped with VSV-G by co-transfecting 293T cells with a VSV-G expression plasmid (Clontech, Takara Bio USA, Mountain View, CA, USA) at a 10:1 HIV/VSV-G plasmid ratio. Supernatants were collected 48 h after transfection, ultracentrifuged (17,000× *g* for 1 h at 4 °C), and then the viruses were resuspended in PBS 1X and stored at −80 °C.

#### 4.5.3. CPRG and MTT Assay: On HelaP4 Cells

HelaP4 cells (10^4^ cells/well) were infected with viruses (7.5 ng p24) in the presence of increasing concentration of compounds. Cells were incubated for 48 h before analysis. β-Galactosidase is under the control of the HIV-1 LTR. Infectivity is correlated with the number of infected cells. Cells were lysed for 10 min at 37 °C using 80 µL of lysis buffer (Na_2_HPO_4_ 60 mM, NaH_2_PO_4_ 60 mM, KCl 10 mM, MgSO_4_ 10 mM, EDTA 25 mM, and NP-40 0.125%). After lysis, 170 µL of CPRG buffer (phosphate buffer 100 mM, pH 7.4, MgCl_2_ 100 mM, and CPRG 20 mM) was added to each well. The plates were incubated at 37 °C until the color changed to dark red. Finally, a plate reader was used to read the absorbance at 572 nm. The MTT assay varies based on NAD(P)H-dependent cellular oxidoreductase enzymes of viable cells reducing the yellow tetrazolium salt MTT (3-(4,5-dimethylthiazol2-yl)-2,5-diphenyltetrazolium bromide). Spectrophotometric measurement can be used to quantify the insoluble purple formazan product that is produced by this reaction. An amount of 10 µL of MTT reagent (7.5 mg/mL in PBS, pH 7.4) was added to each well of the microtiter plate before incubating at 37 °C for 2 h. After 2 h of incubation, 100 µL of MTT lysis buffer (SDS 10%, HCl 0.01 N, pH 4.7) was added and the resulting optical density (OD) readings were measured at 572 nm.

#### 4.5.4. Fluorescence-Activated Cell Sorting (FACS) Combined with Zombie Aqua

MT4 cells (10^5^ cells/well) were infected with viruses (100 ng/well) in the presence of increasing concentration of lawsone compounds and the positive control efavirenz (Sigma-Aldrich, St. Louis, MO, USA). After 48 h, cells from each well were washed with PBS and centrifuged (1500 rpm, 5 min). Then, cells were incubated with Zombie Aqua™ (Biolegend, San Diego, CA, USA) dye solution for 15 min in the dark at room temperature to stain dead cells. After that, the cell pellets were washed one more time with PBS before analysis by flow cytometer. Acquisition was performed on a FACS Celesta (BD Biosciences) and analysis was performed using FlowJo v10 software. Cells were first gated on the FSC-H and FSC-A parameters to exclude doublet cells. Then, living cells were selected according to the Zombie Acqua staining (dead cells are stained whereas living cells are not). Finally, the percentage of HIV-1infected cells was measured by analyzing the percentage of GFP+ cells among living cells. Analysis was performed as reported [32].

At the same time point, cells were harvested and washed with PBS before centrifugation to form pellets. DNA extraction was carried out using the QIAamp DNA Blood Mini Kit (Qiagen, Hilden, Germany). The procedure involved lysing the cells with AL buffer and Proteinase K to release the DNA. The lysate was then mixed with ethanol to facilitate DNA binding to a silica-based membrane in a spin column. The bound DNA was washed with buffers AW1 and AW2 to remove contaminants, and, finally, the DNA was eluted in 100 μL of water. DNA quantification was performed using quantitative polymerase chain reaction (qPCR) with the Roche LightCycler instrument. Accordingly, DNA quantification was conducted using real-time PCR (qPCR) on the same apparatus. This analysis utilized nucleic acid amounts corresponding to approximately 200,000 cells. The sequences of primers and probes employed for qPCR, aimed at absolute quantification of total HIV-1 viral DNA, have been previously described [56]. The copy numbers of total HIV-1 DNA were ascertained from calibration curves derived from amplifying known quantities of cloned DNA with matching sequences.

### 4.6. Inhibition of SARS-CoV-2 Replication

Inhibition of SARS-CoV-2 replication in VEROE6-EGFP cells by compounds **1e** and **2k** was studied as described previously [57]. A SARS-CoV-2 replication assay was conducted in the presence of 2 μM CP100356, a non-toxic inhibitor of the efflux transporter P-gp, which is highly expressed in Vero E6, to not underestimate the effect of the compound due to its depletion from the cell. All experiments were conducted in triplicate.

### 4.7. ADMET Analysis

For ADMET analysis, the SMILES notations of **1e**, **2k**, compound **13**, and RDS1759 were uploaded to SwissADME [58], a free web server to predict the ADMET parameters of a molecule associated with pharmacokinetics, drug likeness, and drug optimization.

## 5. Conclusions

Screening of a series of lawsone Mannich bases led to the identification of **1e** and **2k** as strong HIV-1 RT-associated RNase H inhibitors. **2k** also inhibited HIV-1 replication at low toxicity. Docking calculations revealed the relevance of the naphthoquinone and fatty alkyl moieties of **2k** for target interaction. Compound contacts with enzyme amino acid residues and with RNA nucleobases were observed. Thus, the antiviral activity of **2k** is worth being further explored with the perspective of its development as a cost-effective HIV treatment. In contrast to **2k**, the benzylamino derivative **1e** inhibited SARS-CoV-2 replication, albeit at lower doses and reduced selectivity than a known 3CLpro inhibitor. But the identification of **1e** as a SARS-CoV-2 inhibitor can pave the way for the design of more potent and selective antiviral naphthoquinone-based drugs for the treatment of COVID-19 infections in the future.

## Figures and Tables

**Table 1:** Inhibitory concentrations IC_50_ (in µM) ^1^ of test compounds **1a**–**j** and **2a**–**p** when tested against HIV-1 RT-associated RNase H activity. RDS1759 and compound **13** were applied as positive controls.

Compound	HIV-1 RT RNase H	Compound	HIV-1 RT RNase H
**RDS1759**	8.7 ± 3.1	**2a**	21.5 ± 0.9
**13**	5.8 ± 1.3	**2b**	15.0 ± 5.1
**Lawsone** ^2^	>100	**2c**	7.3 ± 4.7
**1a**	31.2 ± 6.5	**2d**	17.6 ± 3.8
**1b**	4.4 ± 1.1	**2e**	8.5 ± 1.6
**1c**	4.0 ± 0.01	**2f**	7.0 ± 0.9
**1d**	10.7 ± 4.1	**2g**	>100
**1e**	3.1 ± 1.9	**2h**	20.8 ± 0.2
**1f**	17.4 ± 4.1	**2i**	4.7 ± 1.6
**1g**	>100	**2j**	43.7 ± 3.5
**1h**	>100	**2k**	2.8 ± 1.4
**1i**	78.6 ± 11.5	**2l**	12.7 ± 3.4
**1j**	>100	**2m**	7.1 ± 1.1
-	-	**2n**	105.3 ± 10.6
-	-	**2o**	32.5 ± 10.5
-	-	**2p**	19.6 ± 3.4

^1^ Compound concentration required to reduce the HIV-1 RT-associated RNase H activity by 50%. ^2^ Value taken from Min et al. [33].

**Table 2:** Inhibition of SARS-CoV-2 replication in VEROE6-EGFP cells. Effective concentrations EC_50_ for viral replication inhibition (in µM) and cytotoxic concentrations CC_50_ (in µM) ^1^ of **1e**, **2k**, and GC-376 (positive control) in the presence of 2 µM CP-100356 (non-toxic P-gp inhibitor).

Compound	EC_50_	CC_50_	SI ^2^
**GC-376**	0.84 ± 0.08	>100	>119
**1e**	11.2 ± 2.1	35.3 ± 1.3	3.15
**2k**	>41.2	41.2 ± 1.3	-

^1^ Compound concentration reducing the viral replication (EC_50_) or cell viability (CC_50_) by 50%. ^2^ Selectivity index calculated from CC_50_/EC_50_.

## Data Availability

Original data can be obtained from the authors upon reasonable request.

## Supplementary Materials

The following supporting information can be downloaded at: https://www.mdpi.com/article/10.3390/molecules30030495/s1. Tables of drug–RNase H interactions, original ^1^H and ^13^C NMR spectra of new compounds, concentration-dependent RNase H inhibition curves, and molecular docking and MD simulation details: Tables S1 and S2, Figures S1–S25.
