# Supplementary material for: Identification of HIV-1 Reverse Transcriptase-Associated Ribonuclease H Inhibitors Based on 2-Hydroxy-1,4-naphthoquinone Mannich Bases
Source: Molecules. 2025 Jan 23;30(3):495. doi: 10.3390/molecules30030495 (PMC11820915; doi:10.3390/molecules30030495)
Supplement: Supplementary file 1 [file molecules-30-00495-s001.zip › molecules-3326912-supplementary.pdf]

Supplementary Materials

Identification of HIV-1 Reverse Transcriptase-Associated Ribonuclease H Inhibitors Based on 2-Hydroxy-1,4-naphthoquinone Mannich Bases

Nhat Quang Tu [1], Clémence Richetta [1], Federica Putzu [2], Olivier Delelis [1], Khursheed Ahmed [3], Vijay H. Masand [4], Rainer Schobert [5], Enzo Tramontano [2,6], Angela Corona [2,*] and Bernhard Biersack [5,*]

[1] Laboratoire de Biologie et Pharmacologie Appliquée (LBPA), ENS-Paris-Saclay, Centre National de la Recherche Scientifique UMR 8113, Université Paris-Saclay, 91190 Gif-sur-Yvette, France; tunhatquang2@gmail.com (N.Q.T.); clemence.richetta@ens-paris-saclay.fr (C.R.); delelis@lbpa.ens-cachan.fr (O.D.)

[2] Department of Life and Environmental Sciences, University of Cagliari Biomedical Section, Laboratory of Molecular Virology, E Block, First Floor, Cittadella Universitaria di Monserrato SS554, 09042 Monserrato, Italy; f.putzu8@studenti.unica.it (F.P.); tramon@unica.it (E.T.)

[3] Department of Chemistry, Abeda Inamdar Senior College, University of Pune, Pune 411001, India; khursheed92@rediffmail.com

[4] Department of Chemistry, Vidyabharati Mahavidyalaya, Amravati 444602, India; vijaymasand@gmail.com

[5] Organic Chemistry Laboratory, University of Bayreuth, 95447 Bayreuth, Germany; rainer.schobert@uni-bayreuth.de

[6] Istituto di Ricerca Genetica e Biomedica, Consiglio Nazionale delle Ricerche (CNR), 09042 Monserrato, Italy

* Correspondence: angela.corona@unica.it (A.C.); bernhard.biersack@yahoo.com (B.B.)

Table S1. Interactions of **2k** with HIV-1 RT.

LIST OF ATOM-ATOM PROTEIN-LIGAND DISTANCES				LIST OF ATOM-ATOM DNA-LIGAND DISTANCES			
Amino Acid	Atom from Amino acid	Ligand Atom	Distance	Nucleic Acid Base	Atom DNA	Atom Ligand	Distance
ALA445	CA	C	4.891Å	DC803	N1	C	4.975Å
ALA445	CA	H	4.438Å	DC803	N1	H	3.941Å
ALA445	C	C	4.805Å	DC803	C2	C	4.823Å
ALA445	C	H	4.035Å	DC803	C2	H	3.829Å
ALA445	O	C	4.994Å	DC803	O2	H	4.679Å
ALA445	O	H	4.405Å	DC803	N3	C	4.299Å
ALA445	CB	C	4.429Å	DC803	N3	H	3.352Å
ALA445	CB	H	3.670Å	DC803	C4	C	3.909Å
ALA445	HA	H	4.514Å	DC803	C4	H	2.959Å
ALA445	HB1	C	4.958Å	DC803	N4	C	4.001Å
ALA445	HB1	H	4.535Å	DC803	N4	H	3.288Å
ALA445	HB2	C	4.859Å	DC803	C5	C	4.114Å
ALA445	HB2	H	4.816Å	DC803	C5	H	3.127Å
ALA445	HB3	C	3.981Å	DC803	C6	C	4.646Å
ALA445	HB3	H	4.773Å	DC803	C6	H	3.620Å
ALA446	N	C	4.791Å	DC803	H5	C	4.306Å
ALA446	N	H	3.945Å	DC803	H5	H	3.462Å
ALA446	CA	C	4.426Å	DC803	H6	H	4.244Å
ALA446	CA	H	3.432Å	DC803	H41	C	4.158Å
ALA446	C	C	4.305Å	DC803	H41	O	4.987Å
ALA446	C	H	3.483Å	DC803	H41	H	3.559Å
ALA446	O	C	4.959Å	DC803	H42	C	4.320Å
ALA446	O	H	4.341Å	DC803	H42	H	3.728Å
ALA446	CB	H	4.723Å	DC803	H2''	C	4.862Å
ALA446	H	C	4.266Å	DC803	H2''	H	4.064Å
ALA446	H	H	4.652Å	DA804	C5	C	4.934Å
ALA446	HA	C	4.902Å	DA804	C5	H	4.443Å
ALA446	HA	H	4.200Å	DA804	C6	C	4.675Å
ALA446	HB3	H	4.798Å	DA804	C6	H	4.487Å
ASN447	N	C	3.966Å	DA804	N6	C	4.815Å
ASN447	N	H	4.928Å	DA804	N6	H	4.742Å
ASN447	CA	C	4.565Å	DA804	N7	C	4.435Å
ASN447	CA	H	4.082Å	DA804	N7	H	3.701Å
ASN447	CB	C	4.256Å	DA804	C8	H	4.807Å
ASN447	CB	H	4.600Å	DA804	H61	C	4.163Å
ASN447	CG	H	4.390Å	DA804	H61	H	3.894Å
ASN447	ND2	H	4.202Å	DA804	H62	C	4.666Å

ASN447	H	C	4.689Å	DA804	H62	H	4.982Å
ASN447	H	H	4.123Å	DG805	O6	H	4.697Å
ASN447	HA	C	4.645Å	DG721	H2'	O	4.902Å
ASN447	HA	H	4.308Å	DA722	P	CL	3.830Å
ASN447	HB2	C	4.671Å	DA722	C5'	CL	4.445Å
ASN447	HB2	H	4.951Å	DA722	O5'	C	4.641Å
ASN447	HB3	C	4.367Å	DA722	O5'	CL	3.400Å
ASN447	HB3	H	4.406Å	DA722	O5'	H	4.674Å
ASN447	HD21	H	4.834Å	DA722	C4'	CL	4.738Å
ASN447	HD22	C	4.395Å	DA722	C3'	O	4.989Å
ASN447	HD22	H	4.736Å	DA722	C3'	C	4.765Å
THR450	OG1	H	4.675Å	DA722	C3'	CL	3.819Å
THR450	HG1	H	4.619Å	DA722	C3'	H	3.684Å
LEU452	N	H	4.769Å	DA722	O3'	CL	4.576Å
LEU452	CA	C	4.870Å	DA722	O3'	H	4.880Å
LEU452	CA	H	4.649Å	DA722	C2'	C	4.783Å
LEU452	C	C	4.770Å	DA722	C2'	O	3.882Å
LEU452	C	H	4.768Å	DA722	C2'	CL	4.337Å
LEU452	O	C	3.711Å	DA722	C2'	N	4.818Å
LEU452	O	H	4.819Å	DA722	C2'	H	4.731Å
LEU452	CB	C	3.759Å	DA722	C1'	O	4.858Å
LEU452	CB	H	3.338Å	DA722	C1'	H	4.792Å
LEU452	CG	C	4.518Å	DA722	C5	O	4.478Å
LEU452	CG	H	3.903Å	DA722	N6	H	4.988Å
LEU452	CD1	C	4.080Å	DA722	N7	O	4.469Å
LEU452	CD1	H	3.260Å	DA722	N7	C	4.848Å
LEU452	CD2	H	4.809Å	DA722	N7	H	4.721Å
LEU452	H	H	4.877Å	DA722	C8	O	3.853Å
LEU452	HA	H	4.418Å	DA722	C8	C	4.517Å
LEU452	HB2	C	3.640Å	DA722	C8	H	4.407Å
LEU452	HB2	H	3.050Å	DA722	N9	O	4.600Å
LEU452	HB3	C	2.941Å	DA722	N9	H	4.964Å
LEU452	HB3	H	2.763Å	DA722	OP1	CL	4.141Å
LEU452	HG	H	4.725Å	DA722	OP2	C	4.700Å
LEU452	HD11	C	4.017Å	DA722	OP2	CL	3.512Å
LEU452	HD11	H	3.031Å	DA722	OP2	H	4.513Å
LEU452	HD12	C	3.323Å	DA722	H8	O	3.199Å
LEU452	HD12	H	2.638Å	DA722	H8	C	4.108Å
LEU452	HD13	H	4.201Å	DA722	H8	H	4.650Å
LEU452	HD21	C	4.655Å	DA722	H2'	C	4.689Å
LEU452	HD21	H	4.436Å	DA722	H2'	O	3.811Å
GLY453	N	C	4.691Å	DA722	H2'	CL	4.653Å
GLY453	N	H	4.981Å	DA722	H2'	N	3.984Å
GLY453	CA	C	4.837Å	DA722	H2'	H	3.739Å
GLY453	CA	H	4.569Å	DA722	H3'	O	4.822Å
GLY453	C	C	4.706Å	DA722	H3'	C	3.875Å

GLY453	C	H	4.442Å	DA722	H3'	CL	2.766Å
GLY453	O	C	4.754Å	DA722	H3'	H	3.175Å
GLY453	O	H	3.915Å	DA722	H5'	CL	4.551Å
GLY453	H	C	4.627Å	DA722	H61	O	4.614Å
GLY453	H	H	4.984Å	DA722	H61	H	4.353Å
GLY453	HA2	C	4.425Å	DA722	H2''	O	3.007Å
GLY453	HA2	H	4.614Å	DA722	H2''	C	4.183Å
GLY453	HA3	C	4.954Å	DA722	H2''	CL	3.911Å
GLY453	HA3	H	4.032Å	DA722	H2''	N	4.686Å
LYS454	N	C	4.846Å	DA722	H2''	H	2.940Å
LYS454	N	H	4.540Å	DC723	P	C	4.837Å
LYS454	CA	H	4.921Å	DC723	P	CL	4.438Å
LYS454	CB	H	4.417Å	DC723	P	N	4.993Å
LYS454	CG	C	4.082Å	DC723	P	H	4.389Å
LYS454	CG	H	4.873Å	DC723	C5'	C	4.366Å
LYS454	CD	C	4.346Å	DC723	C5'	H	4.733Å
LYS454	CD	H	4.478Å	DC723	O5'	C	4.175Å
LYS454	CE	C	4.584Å	DC723	O5'	H	3.312Å
LYS454	CE	H	4.414Å	DC723	C4'	C	4.720Å
LYS454	NZ	C	4.561Å	DC723	C4'	H	3.784Å
LYS454	NZ	H	4.110Å	DC723	O4'	H	4.445Å
LYS454	H	C	4.941Å	DC723	C3'	C	3.976Å
LYS454	H	H	4.970Å	DC723	C3'	H	4.900Å
LYS454	HA	H	4.924Å	DC723	O3'	C	4.347Å
LYS454	HB2	C	4.778Å	DC723	O3'	H	4.782Å
LYS454	HB2	H	4.741Å	DC723	C2'	C	4.125Å
LYS454	HB3	H	4.952Å	DC723	C2'	H	4.492Å
LYS454	HG2	C	4.834Å	DC723	C1'	H	4.542Å
LYS454	HG2	H	4.870Å	DC723	N1	H	4.394Å
LYS454	HG3	C	4.294Å	DC723	C4	O	4.744Å
LYS454	HG3	H	4.670Å	DC723	C4	C	4.194Å
LYS454	HD2	H	4.329Å	DC723	C4	H	4.713Å
LYS454	HD3	C	4.624Å	DC723	N4	O	4.545Å
LYS454	HD3	H	4.665Å	DC723	N4	C	3.310Å
LYS454	HE2	C	3.765Å	DC723	N4	H	3.433Å
LYS454	HE2	H	4.590Å	DC723	C5	O	3.950Å
LYS454	HE3	C	4.394Å	DC723	C5	C	4.282Å
LYS454	HE3	H	4.027Å	DC723	C5	N	3.840Å
LYS454	HZ1	C	4.792Å	DC723	C5	H	4.288Å
LYS454	HZ1	H	4.280Å	DC723	C6	C	4.411Å
LYS454	HZ2	H	4.729Å	DC723	C6	O	4.832Å
LYS454	HZ3	C	4.470Å	DC723	C6	N	3.937Å
LYS454	HZ3	H	4.952Å	DC723	C6	H	4.928Å
PRO468	CB	H	4.926Å	DC723	OP1	CL	4.736Å
PRO468	HA	H	4.637Å	DC723	OP1	C	4.971Å
PRO468	HB2	C	4.961Å	DC723	OP1	H	4.218Å

PRO468	HB2	H	4.592Å	DC723	OP2	O	4.947Å
PRO468	HB3	C	4.734Å	DC723	OP2	C	4.246Å
PRO468	HB3	H	4.236Å	DC723	OP2	CL	3.575Å
LEU469	N	H	4.994Å	DC723	OP2	N	3.698Å
LEU469	O	H	4.346Å	DC723	OP2	H	4.563Å
LEU469	H	H	4.237Å	DC723	H5	O	4.376Å
ASN474	OD1	H	4.745Å	DC723	H5	C	3.657Å
ASN474	HD22	H	4.591Å	DC723	H5	N	3.129Å
PRO537	CA	H	4.812Å	DC723	H5	H	4.954Å
PRO537	C	H	4.468Å	DC723	H6	C	4.752Å
PRO537	CB	H	4.910Å	DC723	H6	O	4.745Å
PRO537	HA	H	4.371Å	DC723	H6	N	3.316Å
PRO537	HB2	CL	4.200Å	DC723	H6	H	3.971Å
PRO537	HB2	H	4.319Å	DC723	H2'	C	4.487Å
PRO537	HB3	H	4.967Å	DC723	H2'	H	4.848Å
ALA538	N	C	4.445Å	DC723	H3'	C	4.525Å
ALA538	N	H	3.526Å	DC723	H3'	H	4.257Å
ALA538	CA	CL	4.881Å	DC723	H4'	H	4.833Å
ALA538	CA	C	4.529Å	DC723	H41	O	3.849Å
ALA538	CA	H	3.848Å	DC723	H41	C	3.778Å
ALA538	C	C	4.705Å	DC723	H41	H	3.359Å
ALA538	C	CL	4.681Å	DC723	H5'	C	4.518Å
ALA538	C	H	3.822Å	DC723	H5'	H	3.710Å
ALA538	O	C	4.894Å	DC723	H42	O	3.731Å
ALA538	O	CL	3.532Å	DC723	H42	C	2.452Å
ALA538	O	H	3.259Å	DC723	H42	H	2.794Å
ALA538	CB	C	4.162Å	DC723	H2''	N	4.222Å
ALA538	CB	CL	3.741Å	DC723	H2''	C	4.928Å
ALA538	CB	H	3.645Å	DC723	H2''	H	4.465Å
ALA538	H	C	4.933Å	DC723	H5''	H	4.305Å
ALA538	H	CL	4.980Å	DT724	P	C	4.766Å
ALA538	H	H	2.973Å	DT724	P	H	4.790Å
ALA538	HA	H	4.931Å	DT724	O5'	H	4.736Å
ALA538	HB1	C	4.802Å	DT724	C4	C	4.994Å
ALA538	HB1	CL	3.902Å	DT724	C4	H	4.887Å
ALA538	HB1	H	4.191Å	DT724	O4	C	4.607Å
ALA538	HB2	C	4.256Å	DT724	O4	N	4.886Å
ALA538	HB2	CL	2.778Å	DT724	O4	O	4.092Å
ALA538	HB2	H	2.694Å	DT724	O4	H	4.755Å
ALA538	HB3	C	4.698Å	DT724	C5	H	4.283Å
ALA538	HB3	CL	4.155Å	DT724	C7	N	4.439Å
ALA538	HB3	H	4.380Å	DT724	C7	C	4.954Å
HIS539	N	H	4.962Å	DT724	C7	O	4.800Å
HIS539	O	CL	4.913Å	DT724	C7	H	4.904Å
HIS539	CB	C	4.851Å	DT724	OP1	C	4.031Å
HIS539	CB	H	4.606Å	DT724	OP1	H	4.645Å

HIS539	CG	C	4.517Å	DT724	OP2	C	4.717Å
HIS539	CG	H	4.299Å	DT724	OP2	H	4.926Å
HIS539	CD2	C	3.970Å	DT724	H6	H	4.979Å
HIS539	CD2	CL	4.204Å	DT724	H71	N	4.195Å
HIS539	CD2	N	4.899Å	DT724	H71	C	3.977Å
HIS539	CD2	H	4.799Å	DT724	H71	H	3.848Å
HIS539	ND1	C	4.335Å	DT724	H72	C	4.316Å
HIS539	ND1	H	4.928Å	DT724	H72	H	4.335Å
HIS539	CE1	C	4.091Å	DT724	H73	C	4.964Å
HIS539	CE1	H	4.179Å	DT724	H73	N	3.664Å
HIS539	NE2	C	4.372Å	DT724	H73	O	3.716Å
HIS539	NE2	CL	4.502Å	DT724	H73	H	4.596Å
HIS539	NE2	H	4.715Å	DG725	H1	H	4.528Å
HIS539	HB2	H	4.995Å	DG725	H21	H	4.664Å
HIS539	HB3	C	4.163Å				
HIS539	HB3	CL	4.078Å				
HIS539	HB3	O	4.571Å				
HIS539	HB3	H	4.784Å				
HIS539	HD2	C	4.371Å				
HIS539	HD2	CL	4.673Å				
HIS539	HD2	O	4.641Å				
HIS539	HD2	N	4.377Å				
HIS539	HD2	H	4.579Å				
HIS539	HE1	C	4.981Å				
HIS539	HE1	H	4.359Å				
HIS539	HE2	C	4.146Å				
HIS539	HE2	CL	4.103Å				
HIS539	HE2	N	4.982Å				
HIS539	HE2	H	4.710Å				
LYS540	CG	CL	4.675Å				
LYS540	CD	CL	4.794Å				
LYS540	CE	CL	3.784Å				
LYS540	CE	H	4.795Å				
LYS540	NZ	CL	4.701Å				
LYS540	HG2	CL	3.946Å				
LYS540	HG3	CL	4.714Å				
LYS540	HE2	CL	3.666Å				
LYS540	HE3	C	4.438Å				
LYS540	HE3	CL	2.944Å				
LYS540	HE3	O	4.702Å				
LYS540	HE3	H	3.839Å				
LYS540	HZ3	CL	4.435Å				
LYS540	HZ3	H	4.622Å				
VAL552	C	C	4.877Å				
VAL552	C	H	4.845Å				
VAL552	O	C	4.830Å				

VAL552	O	H	4.015Å				
VAL552	HG22	H	4.672Å				
SER553	N	H	4.811Å				
SER553	CA	C	4.239Å				
SER553	CA	H	3.823Å				
SER553	C	C	4.993Å				
SER553	C	H	4.277Å				
SER553	O	C	4.772Å				
SER553	O	H	4.824Å				
SER553	CB	C	4.673Å				
SER553	CB	H	4.406Å				
SER553	OG	C	4.869Å				
SER553	OG	H	4.572Å				
SER553	HA	C	4.559Å				
SER553	HA	H	4.293Å				
SER553	HB2	C	4.482Å				
SER553	HB2	H	4.476Å				
SER553	HB3	C	4.485Å				
SER553	HB3	H	4.115Å				
SER553	HG	H	4.613Å				
ASN265	ND2	CL	4.724Å				
ASN265	HD21	CL	3.827Å				

Table S2. Interactions of **1e** with HIV-1 RT.

LIST OF ATOM-ATOM PROTEIN-LIGAND DISTANCES				LIST OF ATOM-ATOM DNA-LIGAND DISTANCES			
Amino Acid	Atom from Amino acid	Ligand Atom	Distance	Nucleic Acid Base	Atom DNA	Atom Ligand	Distance
ALA445	N	H	4.565Å	DA722	O3'	H	4.716Å
ALA445	CA	O	4.498Å	DC723	P	C	4.062Å
ALA445	CA	C	4.837Å	DC723	P	H	3.600Å
ALA445	CA	H	4.934Å	DC723	C5'	O	3.956Å
ALA445	C	O	4.569Å	DC723	C5'	C	4.326Å
ALA445	C	C	4.028Å	DC723	C5'	H	3.024Å
ALA445	C	H	3.110Å	DC723	O5'	O	4.698Å
ALA445	O	C	4.438Å	DC723	O5'	C	4.359Å
ALA445	O	H	4.271Å	DC723	O5'	H	3.046Å
ALA445	CB	O	3.982Å	DC723	C4'	O	4.339Å
ALA445	CB	C	4.705Å	DC723	C4'	C	4.775Å
ALA445	CB	H	4.276Å	DC723	C4'	H	4.083Å
ALA445	H	H	4.269Å	DC723	C3'	O	4.363Å
ALA445	HA	O	4.200Å	DC723	C3'	C	4.238Å
ALA445	HA	H	4.360Å	DC723	C3'	H	4.217Å
ALA445	HB1	O	4.408Å	DC723	O3'	O	4.928Å
ALA445	HB1	C	4.705Å	DC723	O3'	C	4.501Å
ALA445	HB1	H	4.050Å	DC723	O3'	H	4.925Å
ALA445	HB2	O	4.653Å	DC723	OP1	C	4.927Å
ALA445	HB2	C	4.965Å	DC723	OP1	H	2.943Å
ALA445	HB2	H	2.934Å	DC723	OP2	C	4.814Å
ALA445	HB3	O	4.342Å	DC723	OP2	H	4.623Å
ALA445	HB3	C	3.764Å	DC723	H3'	O	3.868Å
ALA445	HB3	H	3.554Å	DC723	H3'	C	3.286Å
ALA446	N	O	4.814Å	DC723	H3'	H	3.460Å
ALA446	N	C	4.351Å	DC723	H4'	O	3.989Å
ALA446	N	H	3.650Å	DC723	H4'	C	4.928Å
ALA446	CA	O	4.842Å	DC723	H4'	H	4.328Å
ALA446	CA	C	4.976Å	DC723	H5'	O	2.920Å
ALA446	CA	H	4.990Å	DC723	H5'	C	3.513Å
ALA446	C	O	3.769Å	DC723	H5'	H	2.177Å
ALA446	C	C	4.589Å	DC723	H5''	O	4.593Å
ALA446	C	H	4.388Å	DC723	H5''	C	4.429Å
ALA446	O	O	3.280Å	DC723	H5''	H	3.687Å
ALA446	O	C	4.124Å	DT724	P	O	4.514Å
ALA446	O	H	4.883Å	DT724	P	C	4.377Å
ALA446	H	O	4.599Å	DT724	P	N	4.467Å

ALA446	H	C	4.993Å	DT724	P	H	4.627Å
ALA446	H	H	4.534Å	DT724	C5'	C	4.385Å
ALA446	HA	C	4.091Å	DT724	C5'	H	4.542Å
ALA446	HA	H	3.981Å	DT724	O5'	C	4.390Å
ASN447	N	O	3.960Å	DT724	O5'	N	4.867Å
ASN447	N	C	4.855Å	DT724	O5'	H	4.860Å
ASN447	N	H	4.651Å	DT724	C4'	C	4.665Å
ASN447	CA	O	3.729Å	DT724	C4'	H	4.176Å
ASN447	CA	H	3.776Å	DT724	C3'	C	4.262Å
ASN447	C	O	4.940Å	DT724	C3'	H	3.589Å
ASN447	CB	O	4.249Å	DT724	O3'	C	4.962Å
ASN447	CB	H	3.939Å	DT724	O3'	H	4.073Å
ASN447	CG	O	4.586Å	DT724	C2'	H	4.712Å
ASN447	CG	C	4.457Å	DT724	OP1	O	4.786Å
ASN447	CG	N	4.269Å	DT724	OP1	C	4.225Å
ASN447	CG	H	4.948Å	DT724	OP1	N	4.718Å
ASN447	ND2	O	4.130Å	DT724	OP1	H	3.931Å
ASN447	ND2	C	3.362Å	DT724	OP2	O	4.341Å
ASN447	ND2	N	4.114Å	DT724	OP2	C	3.931Å
ASN447	ND2	H	4.980Å	DT724	OP2	H	4.445Å
ASN447	OD1	C	4.937Å	DT724	H3'	C	4.338Å
ASN447	OD1	H	4.087Å	DT724	H3'	H	4.607Å
ASN447	H	O	4.693Å	DT724	H4'	C	4.992Å
ASN447	H	C	4.947Å	DT724	H4'	H	4.401Å
ASN447	H	H	4.435Å	DT724	H5'	C	3.464Å
ASN447	HA	O	2.816Å	DT724	H5'	N	4.282Å
ASN447	HA	C	4.977Å	DT724	H5'	H	3.499Å
ASN447	HA	N	4.765Å	DT724	H2''	C	4.986Å
ASN447	HA	H	4.751Å	DT724	H2''	H	4.651Å
ASN447	HB2	H	4.996Å	DT724	H5''	C	4.844Å
ASN447	HB3	O	3.865Å	DT724	H5''	H	4.770Å
ASN447	HB3	N	4.350Å	DG725	P	H	4.010Å
ASN447	HB3	C	4.000Å	DG725	OP1	H	4.181Å
ASN447	HB3	H	4.302Å	DG725	OP2	C	4.247Å
ASN447	HD21	O	4.746Å	DG725	OP2	H	3.356Å
ASN447	HD21	C	2.812Å				
ASN447	HD21	N	4.491Å				
ASN447	HD21	H	4.063Å				
ASN447	HD22	C	4.210Å				
ASN447	HD22	O	3.455Å				
ASN447	HD22	N	3.162Å				
ASN447	HD22	H	4.102Å				
ARG448	H	O	4.371Å				
ARG448	H	H	4.618Å				
GLU449	H	H	4.824Å				
GLU449	HG2	H	4.868Å				

GLU449	HG3	H	4.303Å				
LEU452	CA	H	4.913Å				
LEU452	C	C	4.397Å				
LEU452	C	H	3.768Å				
LEU452	O	C	4.560Å				
LEU452	O	H	4.707Å				
LEU452	CB	H	4.954Å				
LEU452	HB2	C	4.710Å				
LEU452	HB2	H	4.192Å				
LEU452	HB3	H	4.692Å				
GLY453	N	C	4.394Å				
GLY453	N	H	3.558Å				
GLY453	CA	C	3.894Å				
GLY453	CA	H	3.120Å				
GLY453	C	C	4.204Å				
GLY453	C	H	3.300Å				
GLY453	O	C	4.873Å				
GLY453	O	H	3.860Å				
GLY453	H	H	4.170Å				
GLY453	HA2	C	4.158Å				
GLY453	HA2	H	4.546Å				
GLY453	HA3	C	4.717Å				
GLY453	HA3	H	4.065Å				
LYS454	N	C	4.275Å				
LYS454	N	H	3.566Å				
LYS454	CA	H	4.517Å				
LYS454	O	H	4.822Å				
LYS454	CB	C	4.849Å				
LYS454	CB	H	4.333Å				
LYS454	CG	C	4.175Å				
LYS454	CG	H	3.593Å				
LYS454	CD	C	4.514Å				
LYS454	CD	H	4.222Å				
LYS454	CE	C	4.741Å				
LYS454	CE	H	4.648Å				
LYS454	H	C	4.041Å				
LYS454	H	H	3.540Å				
LYS454	HA	H	4.933Å				
LYS454	HB2	C	4.530Å				
LYS454	HB2	H	4.232Å				
LYS454	HG2	C	4.898Å				
LYS454	HG2	H	4.166Å				
LYS454	HG3	C	4.111Å				
LYS454	HG3	H	4.293Å				
LYS454	HD3	C	4.760Å				
LYS454	HD3	H	4.105Å				

LYS454	HE2	C	4.602Å				
LYS454	HE2	H	3.561Å				
LYS454	HE3	H	4.772Å				
ASN474	CG	O	4.357Å				
ASN474	CG	H	4.785Å				
ASN474	ND2	O	4.311Å				
ASN474	ND2	H	4.782Å				
ASN474	OD1	O	3.635Å				
ASN474	OD1	C	4.844Å				
ASN474	OD1	H	4.383Å				
ASN474	HD22	O	3.615Å				
ASN474	HD22	C	4.697Å				
ASN474	HD22	H	4.407Å				
HIS539	CG	H	4.801Å				
HIS539	CD2	C	4.894Å				
HIS539	CD2	H	4.088Å				
HIS539	ND1	C	4.700Å				
HIS539	ND1	H	4.207Å				
HIS539	CE1	C	4.796Å				
HIS539	CE1	H	2.937Å				
HIS539	NE2	C	4.886Å				
HIS539	NE2	H	2.788Å				
HIS539	HD2	H	4.692Å				
HIS539	HE1	C	4.093Å				
HIS539	HE1	H	4.564Å				
HIS539	HE2	C	4.326Å				
HIS539	HE2	H	4.434Å				
VAL552	C	C	4.581Å				
VAL552	C	H	4.260Å				
VAL552	O	C	4.532Å				
VAL552	O	H	4.872Å				
VAL552	HB	H	4.793Å				
SER553	N	C	4.754Å				
SER553	N	H	4.974Å				
SER553	CA	C	4.586Å				
SER553	CA	H	4.729Å				
SER553	C	C	4.684Å				
SER553	C	H	4.481Å				
SER553	O	C	4.714Å				
SER553	O	H	4.924Å				
SER553	CB	C	4.859Å				
SER553	CB	O	4.904Å				
SER553	CB	H	3.622Å				
SER553	OG	H	4.875Å				
SER553	HA	C	4.304Å				
SER553	HA	H	4.996Å				

SER553	HB2	C	4.445Å				
SER553	HB2	H	4.753Å				
SER553	HB3	C	4.235Å				
SER553	HB3	N	4.920Å				
SER553	HB3	O	3.831Å				
SER553	HB3	H	4.390Å				

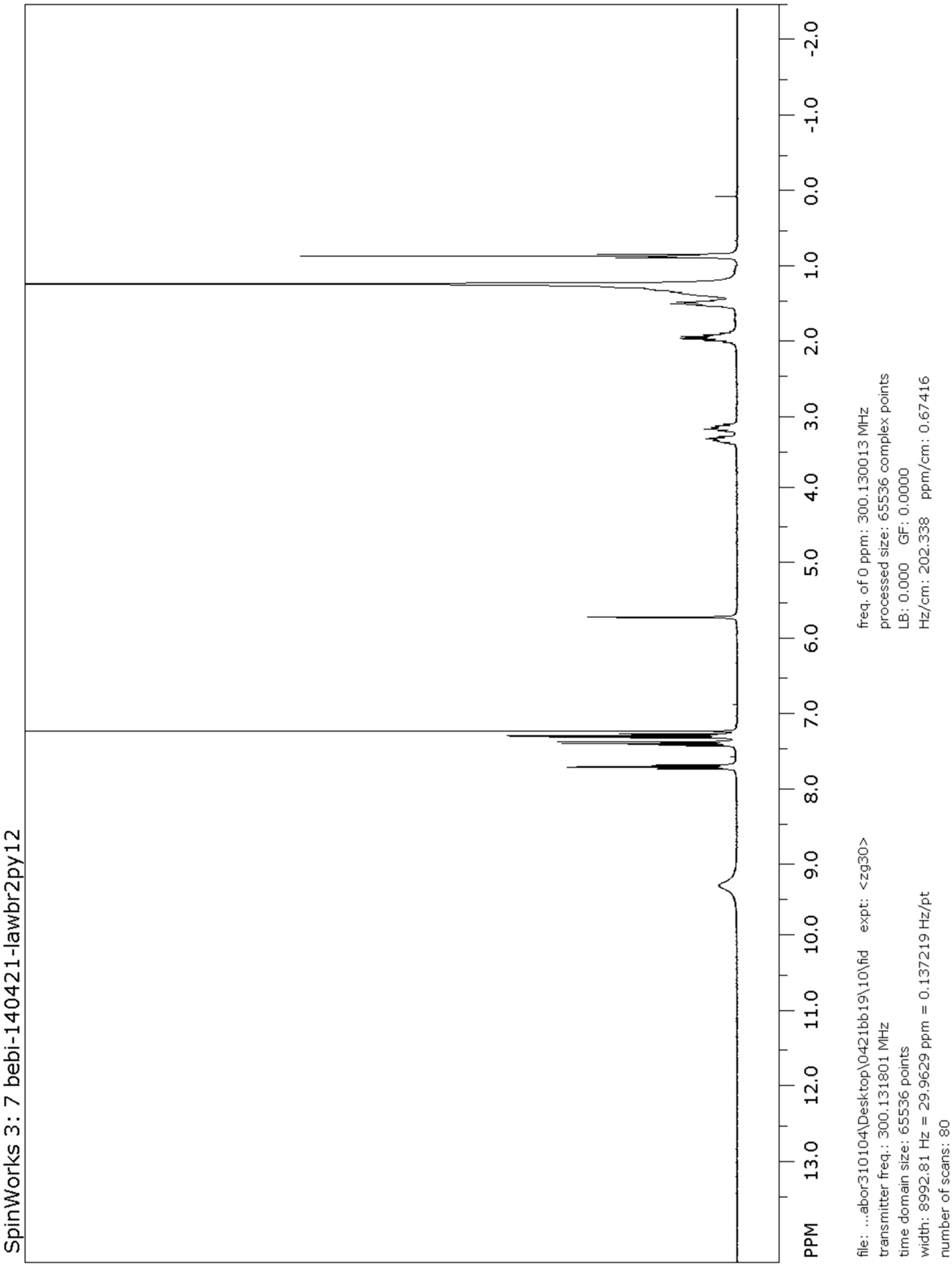

Figure S1. ^{1}H NMR spectrum of **1g**

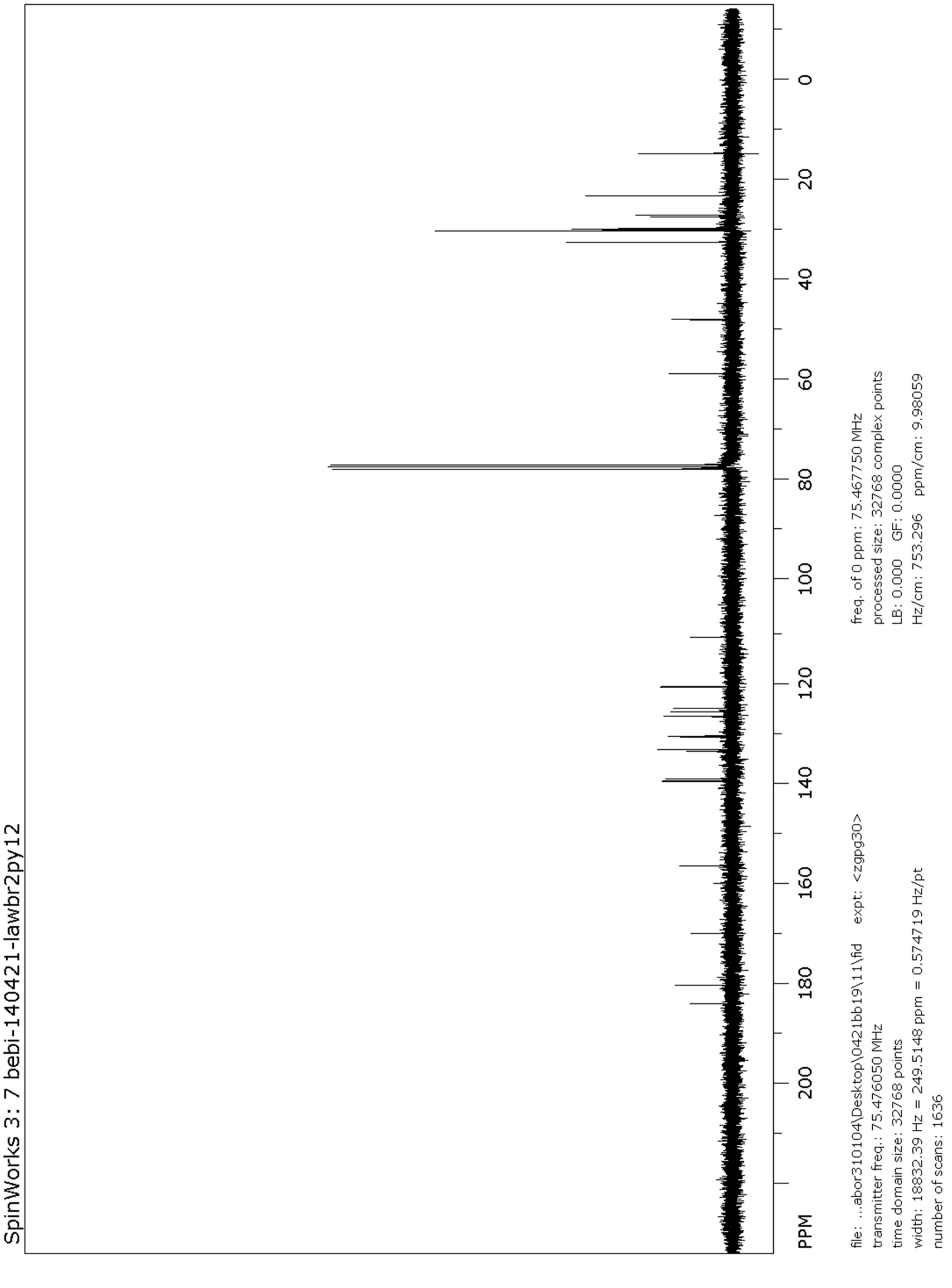

Figure S2. ^{13}C NMR spectrum of **1g**

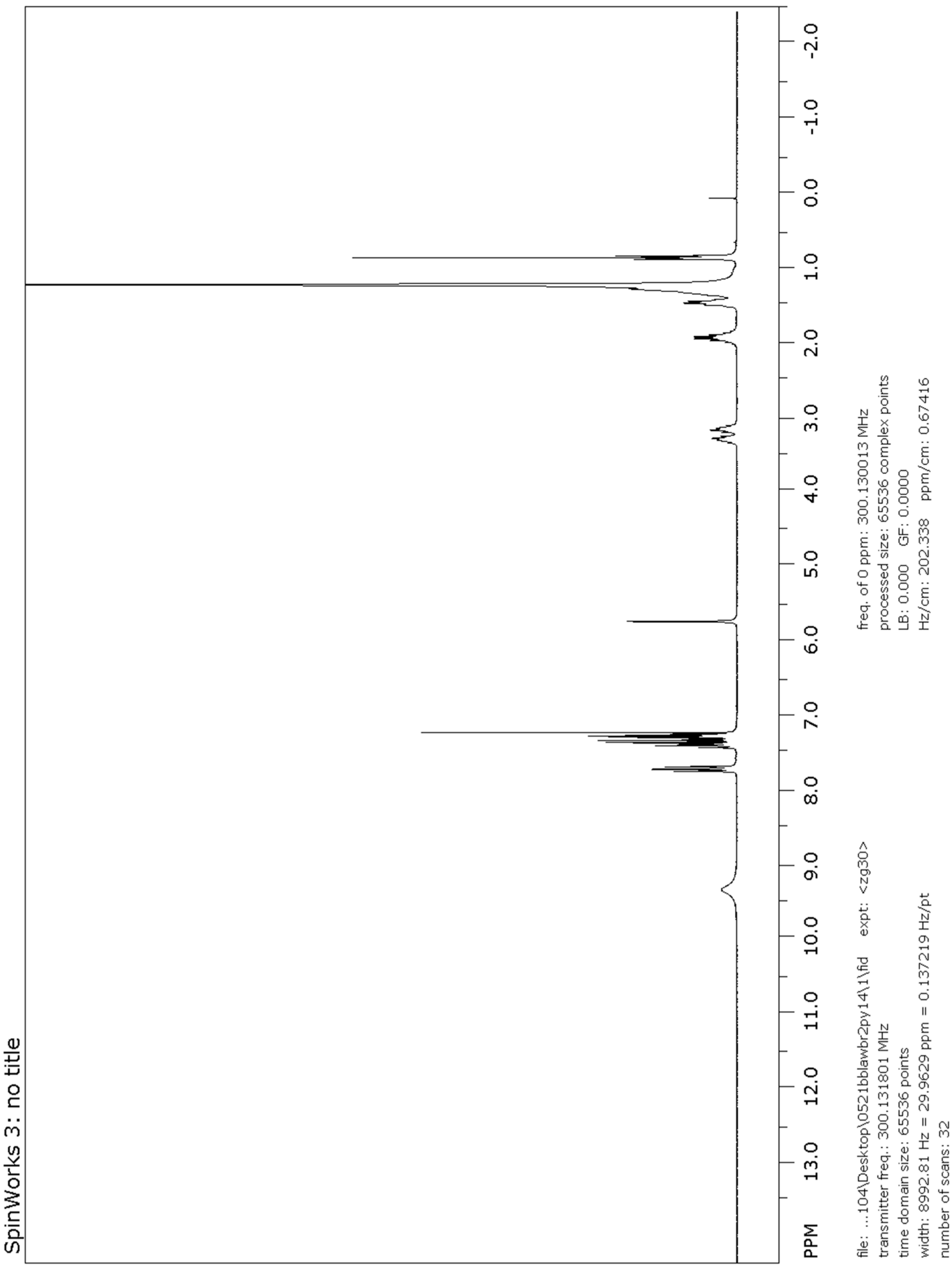

Figure S3. ^{1}H NMR spectrum of **1h**

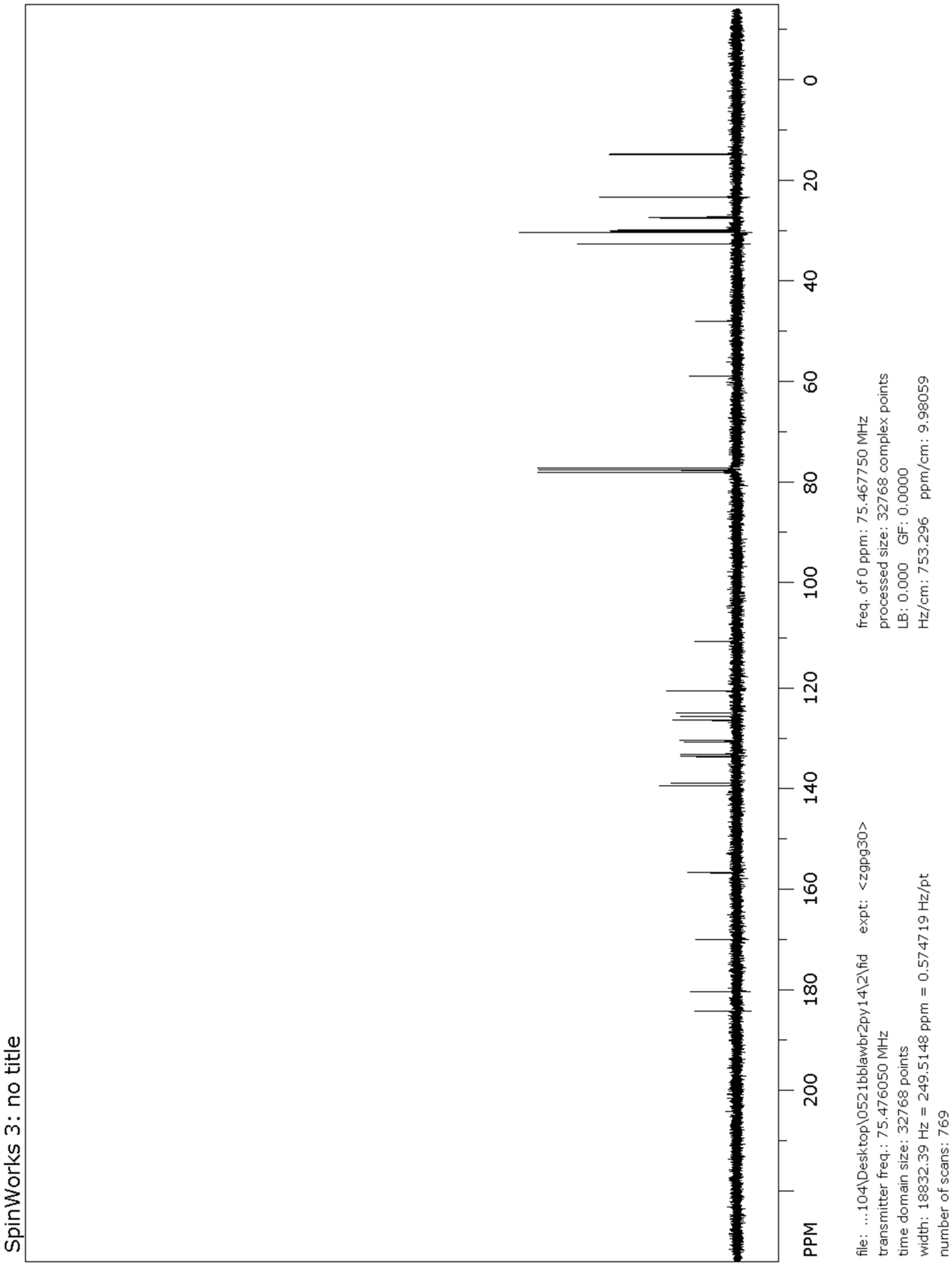

Figure S4. ^{13}C NMR spectrum of **1h**

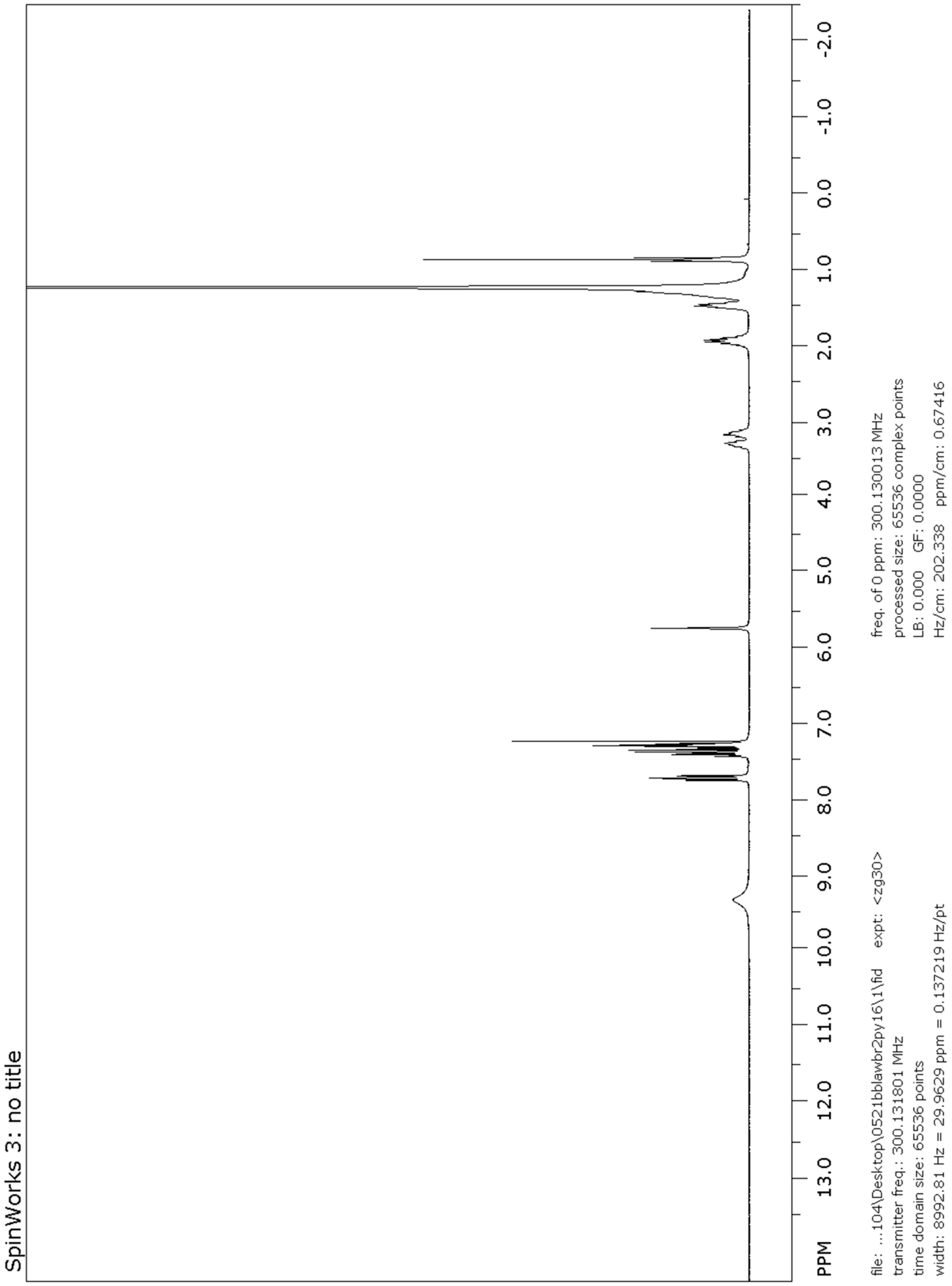

Figure S5. ^{1}H NMR spectrum of **1i**

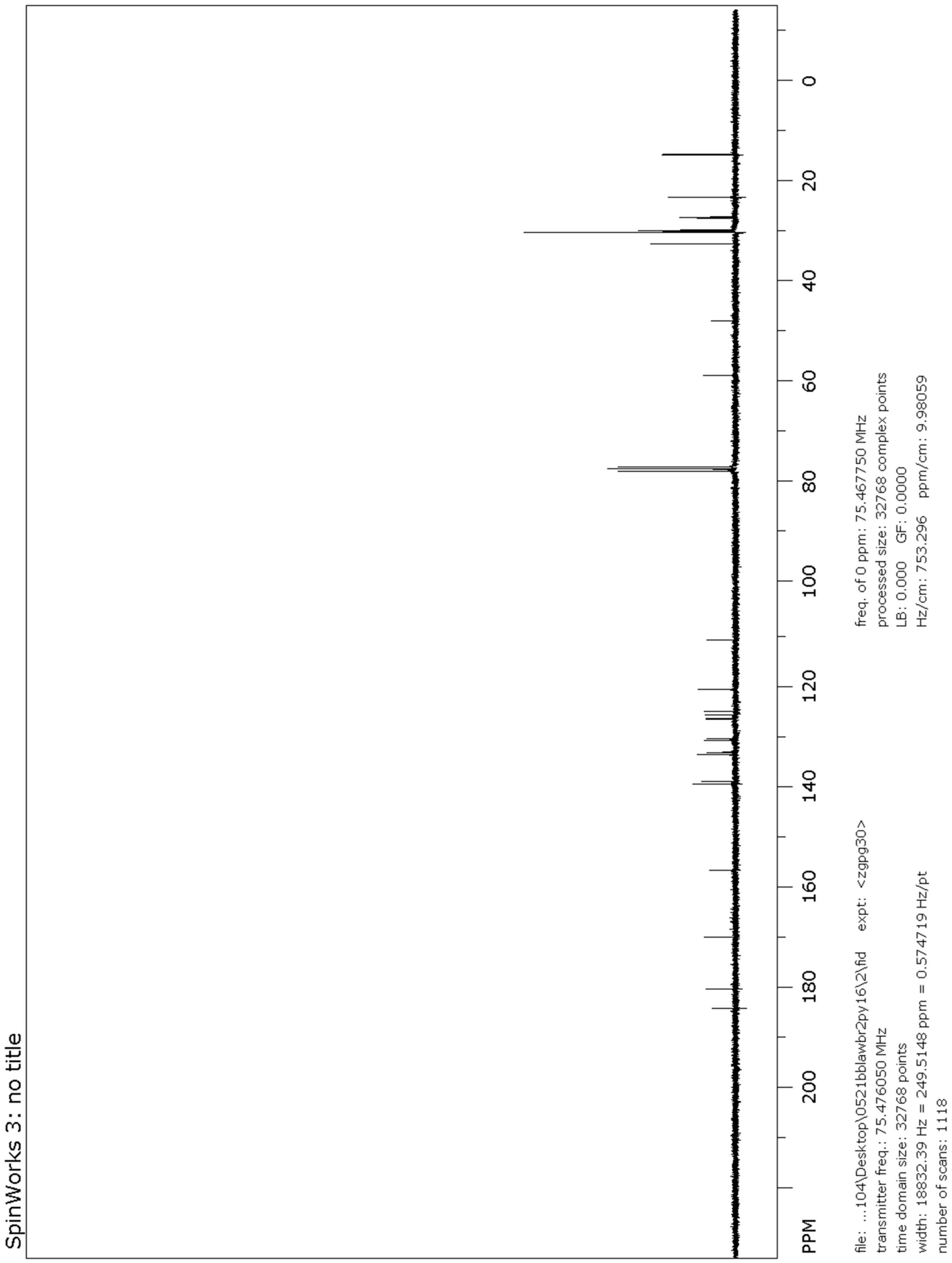

Figure S6. ^{13}C NMR spectrum of **1i**

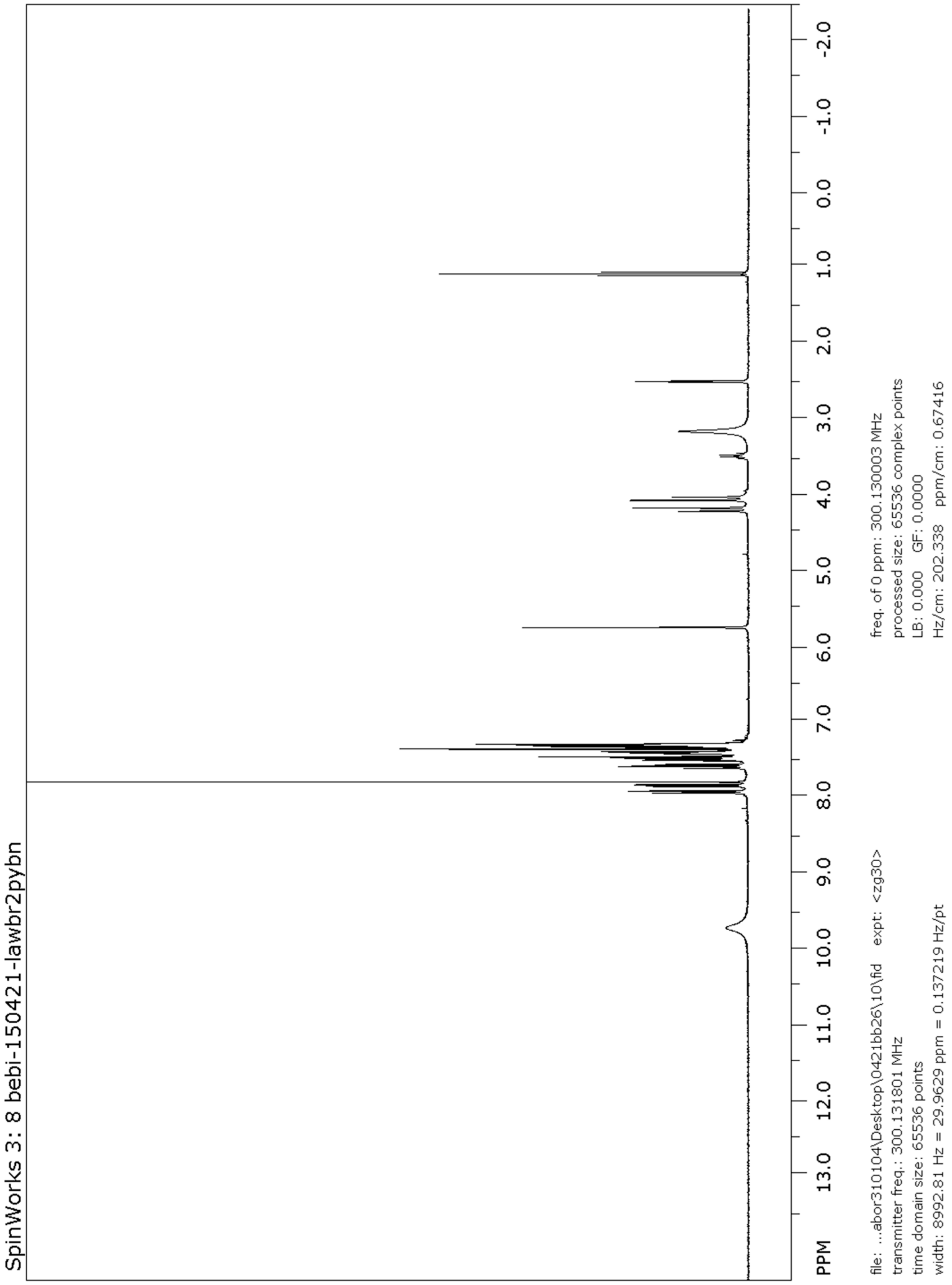

Figure S7. ^{1}H NMR spectrum of **1j**

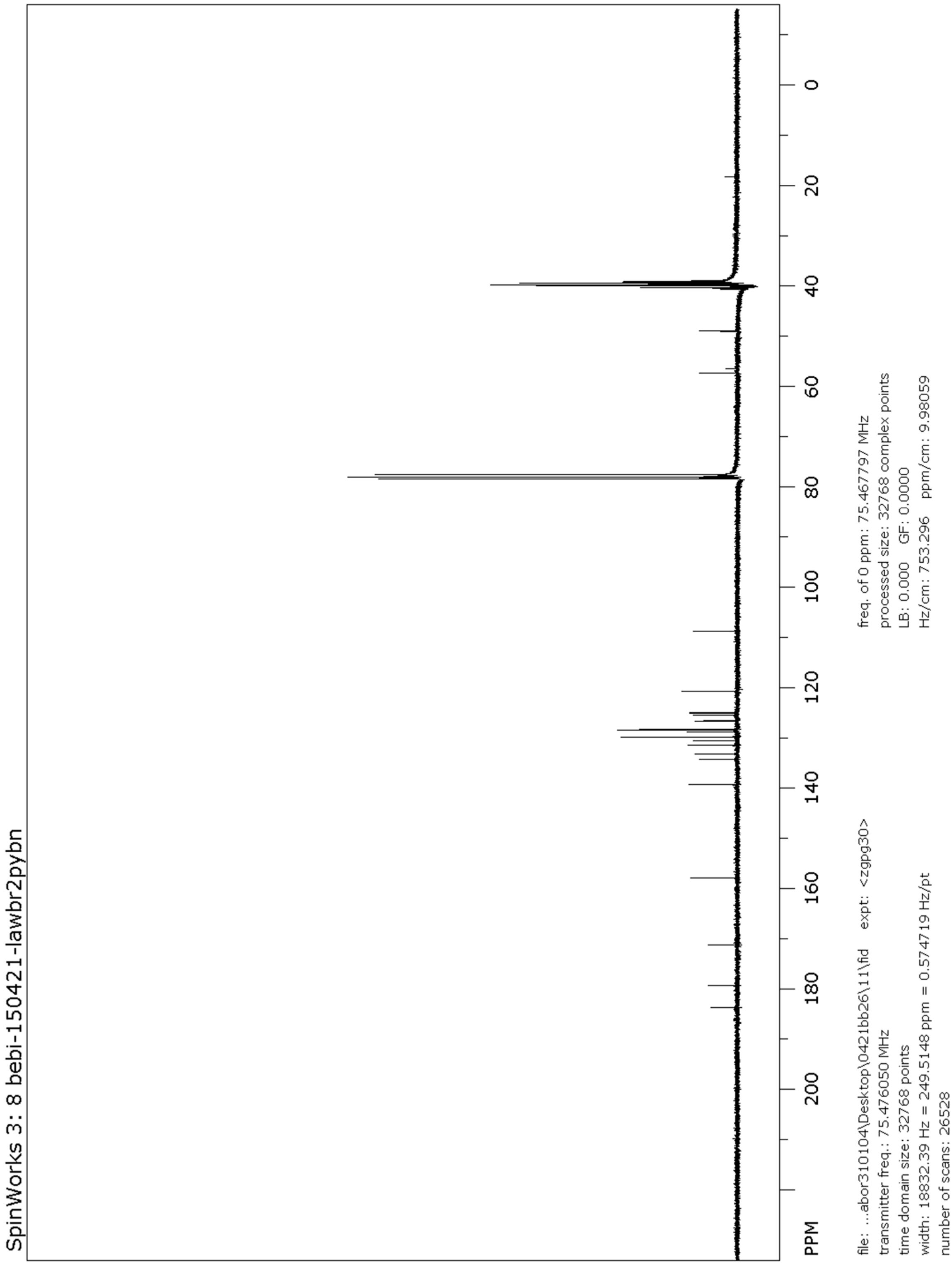

Figure S8. ^{13}C NMR spectrum of **1j**

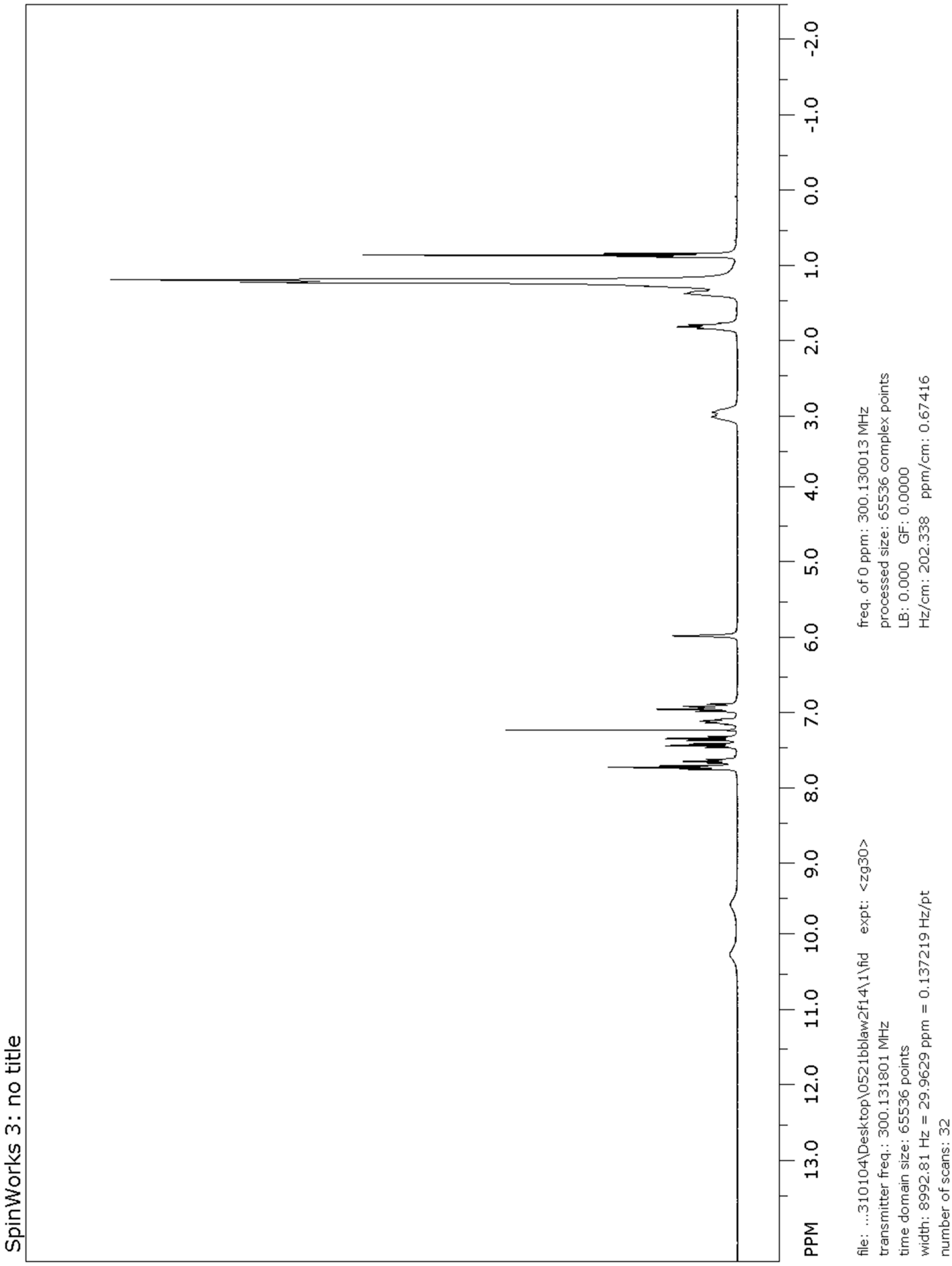

Figure S9. ^{1}H NMR spectrum of **2b**

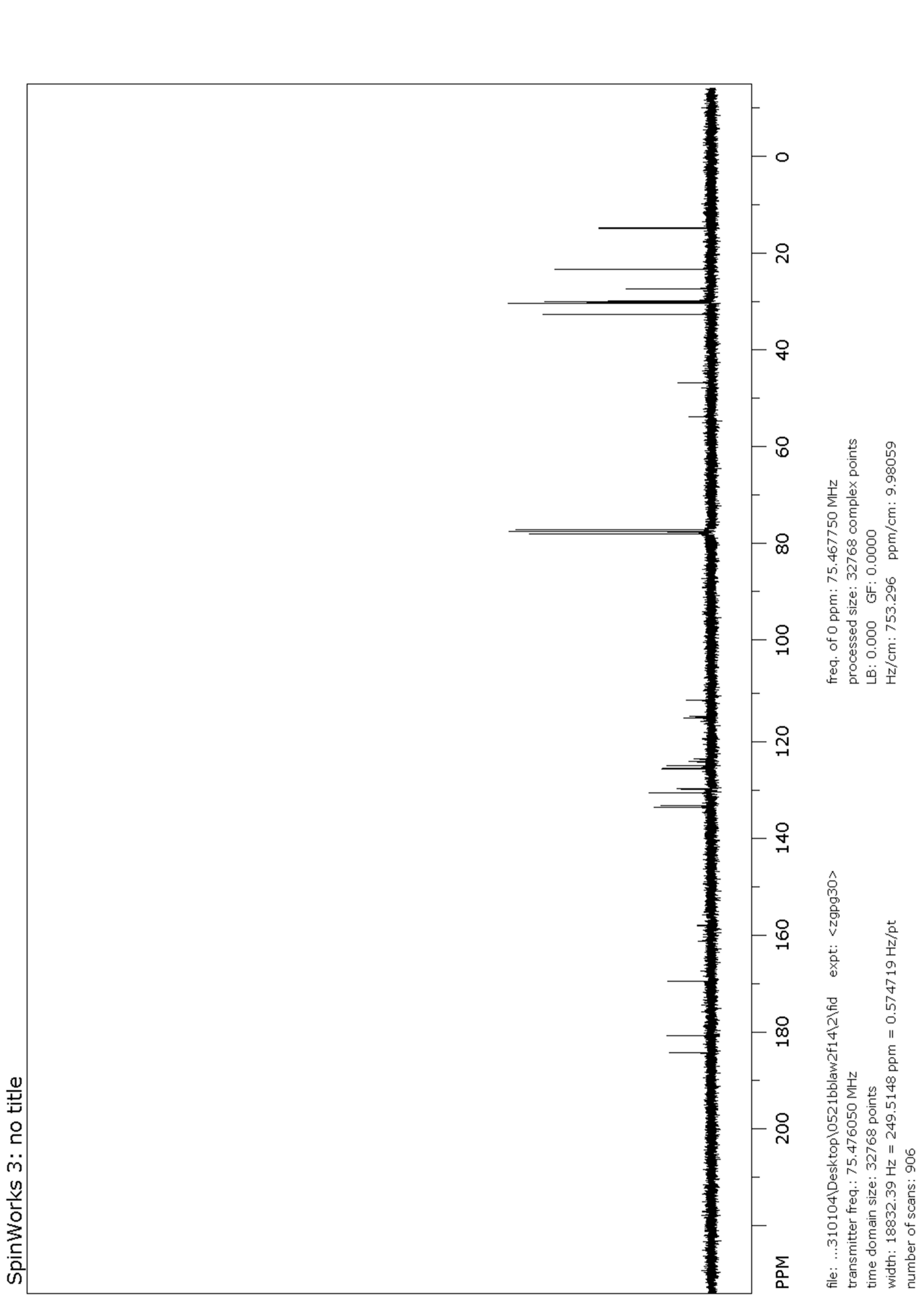

Figure S10. ^{13}C NMR spectrum of **2b**

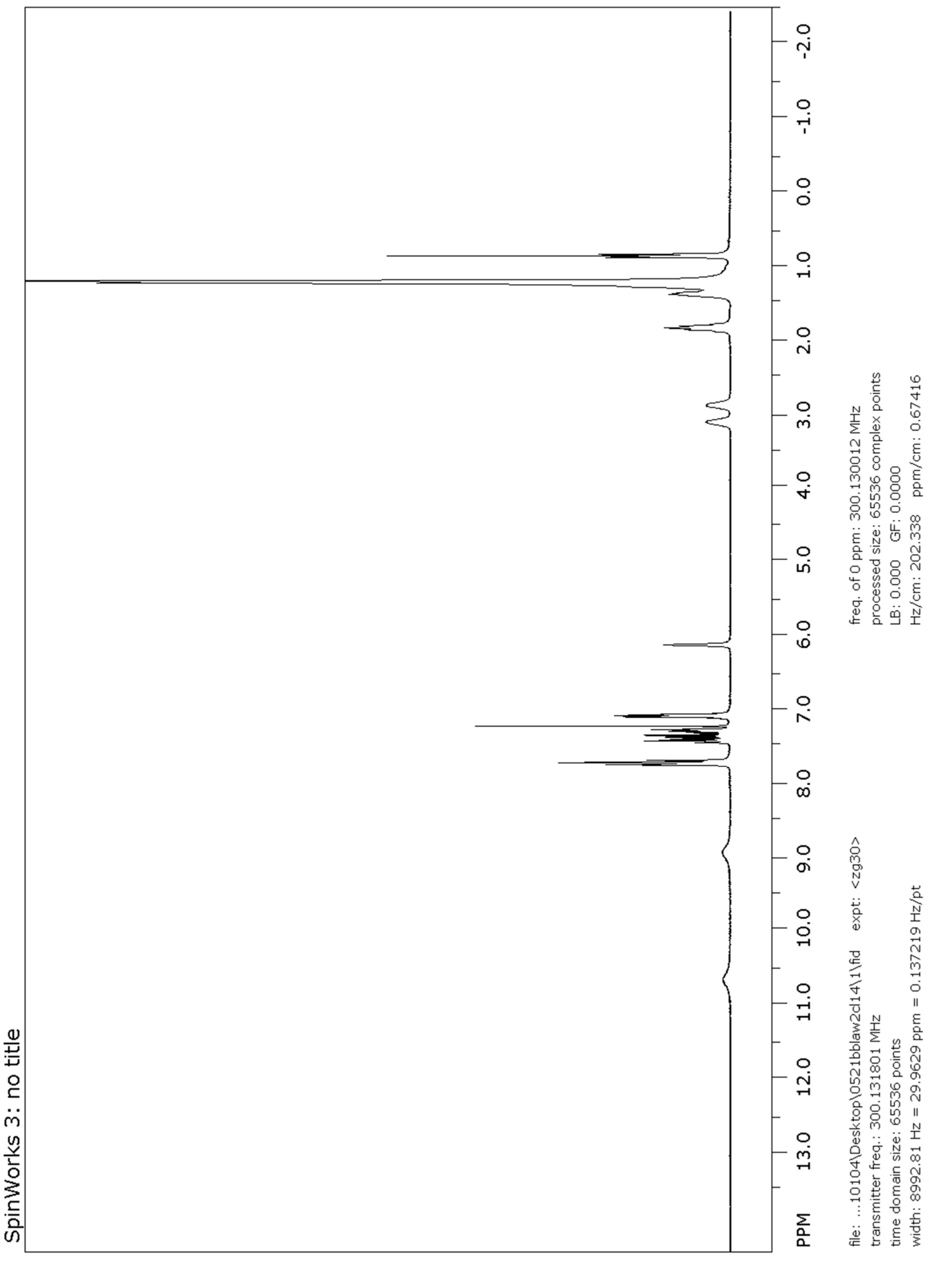

Figure S11. ^{1}H NMR spectrum of **2c**

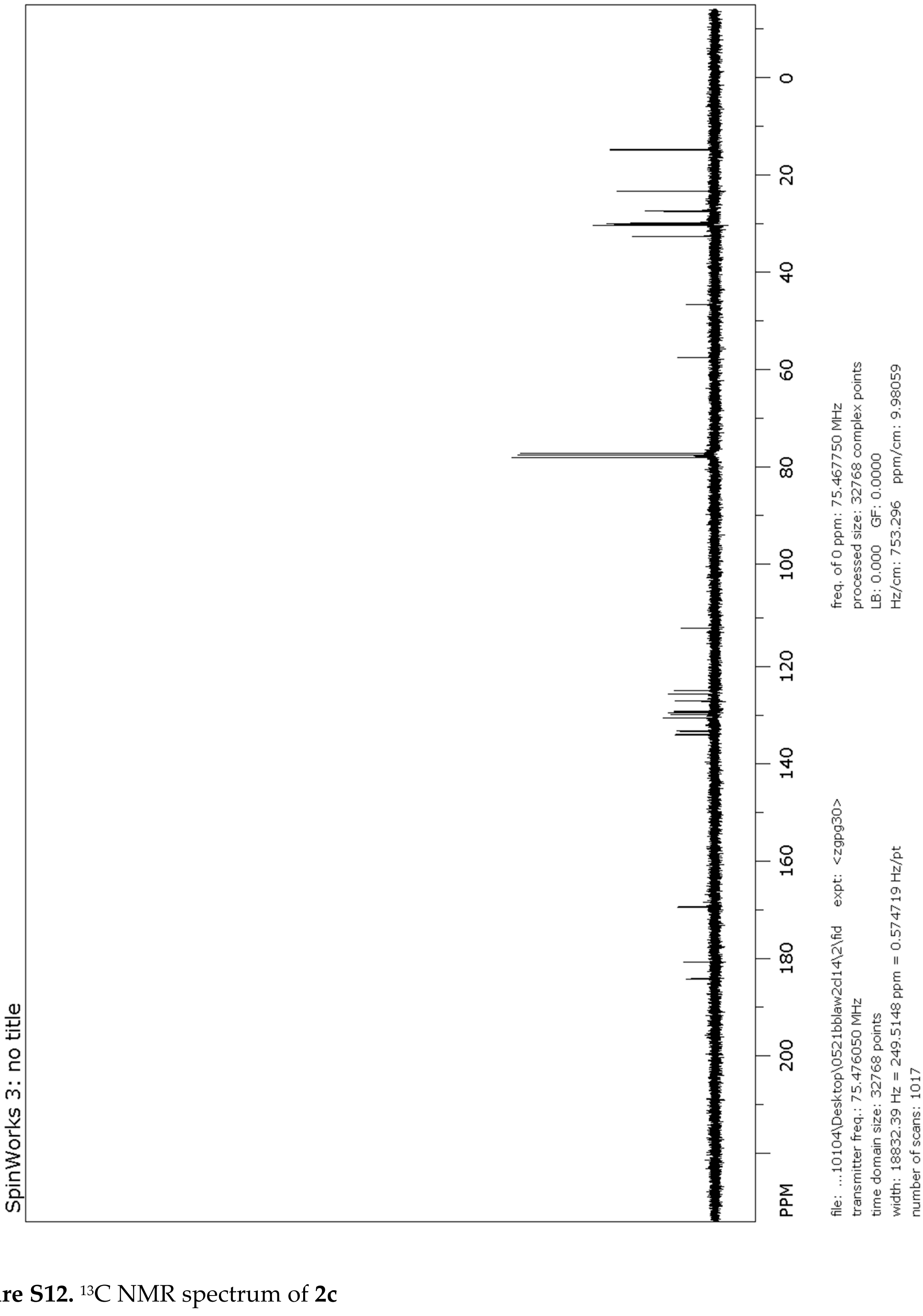

Figure S12. ^{13}C NMR spectrum of **2c**

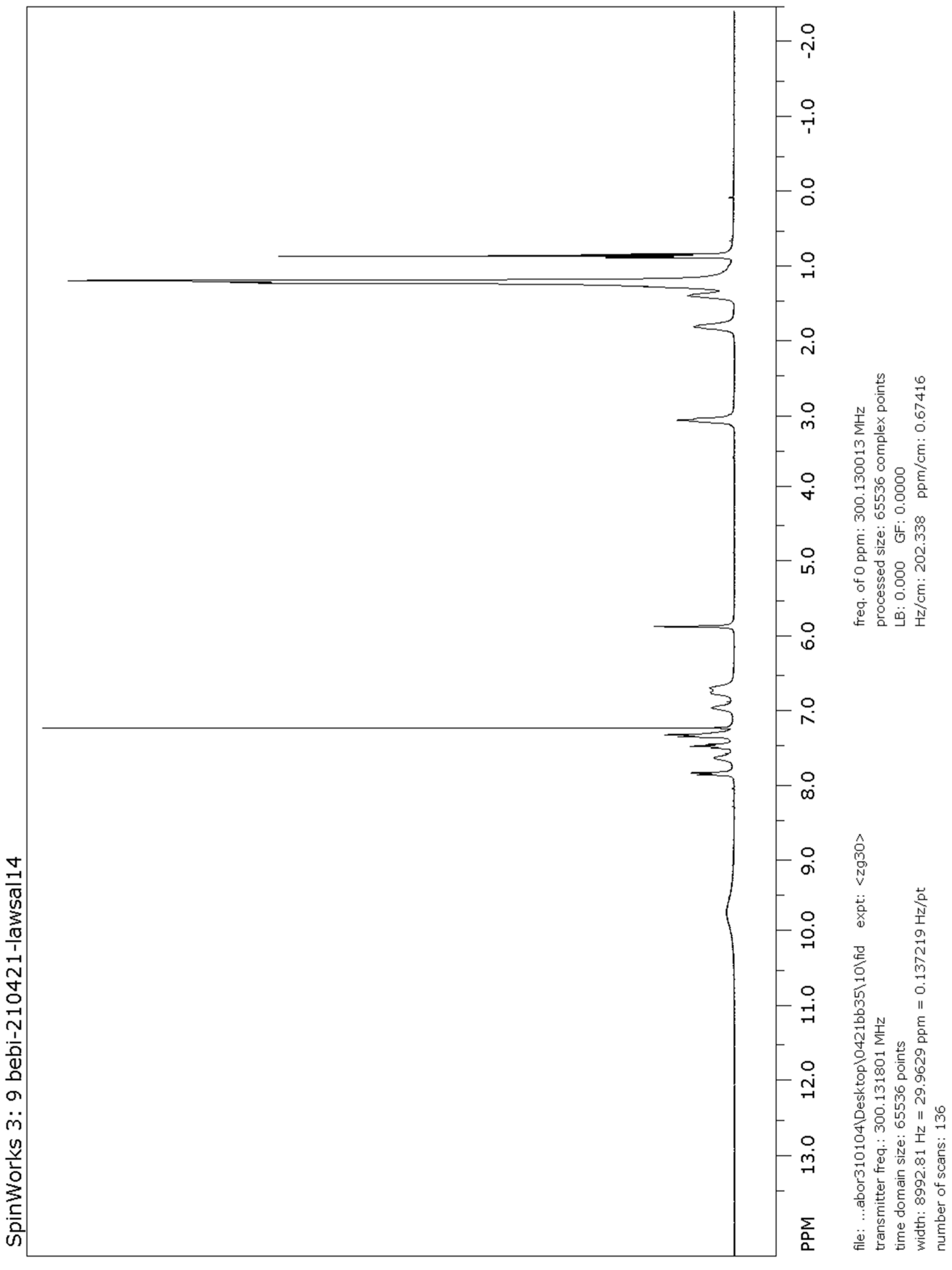

Figure S13. ^{1}H NMR spectrum of **2e**

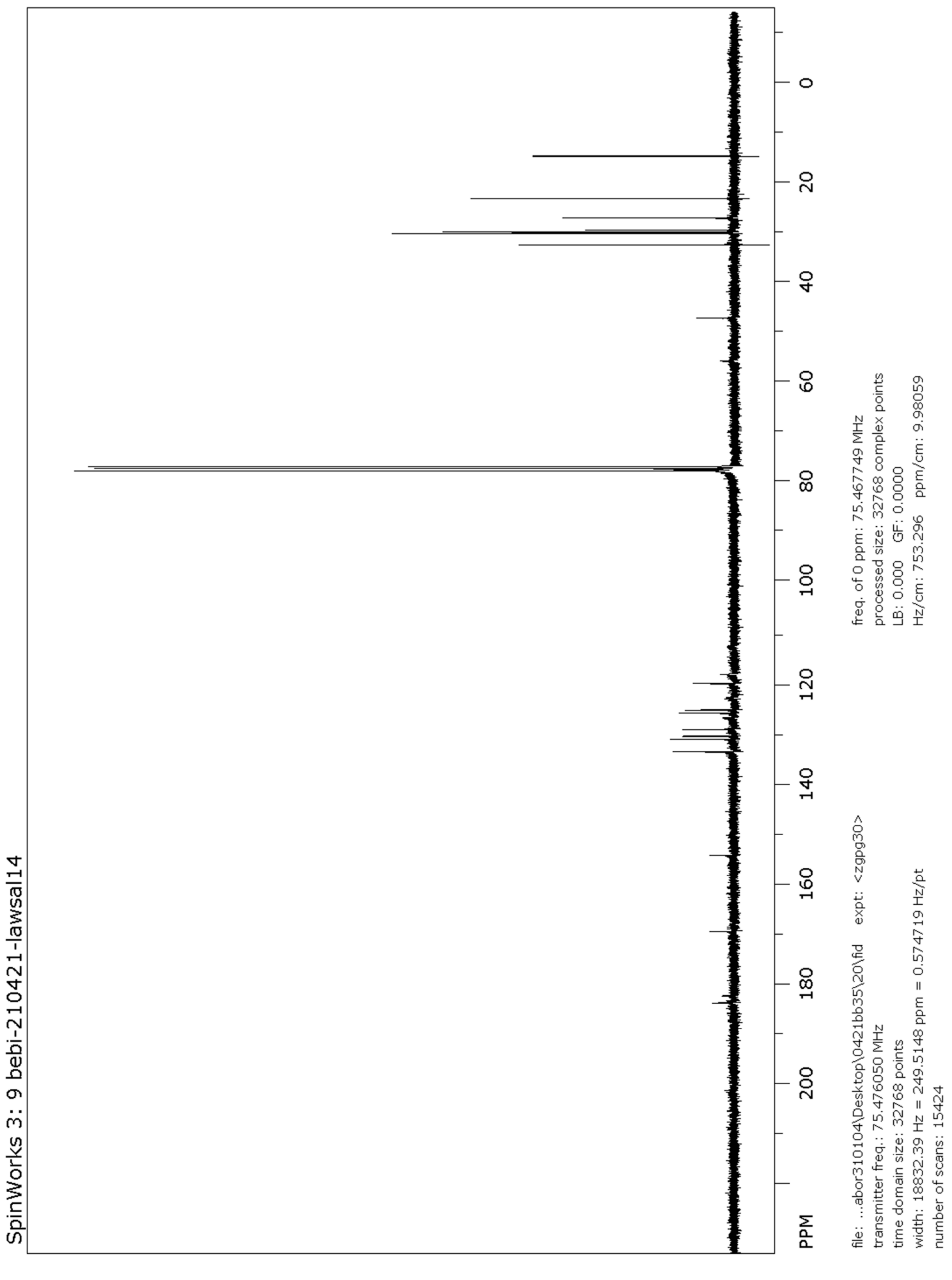

Figure S14. ^{13}C NMR spectrum of **2e**

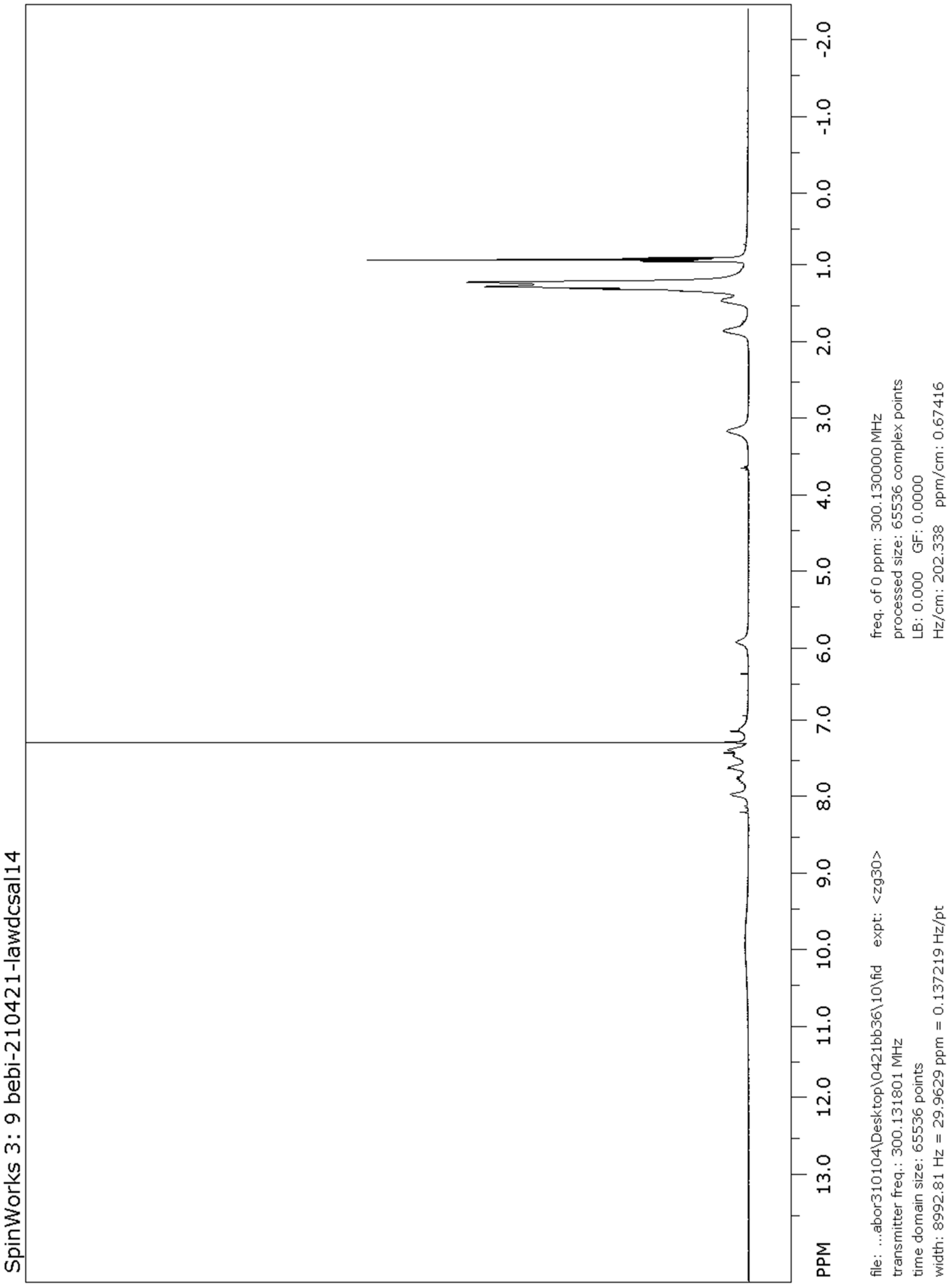

Figure S15. ^{1}H NMR spectrum of **2j**

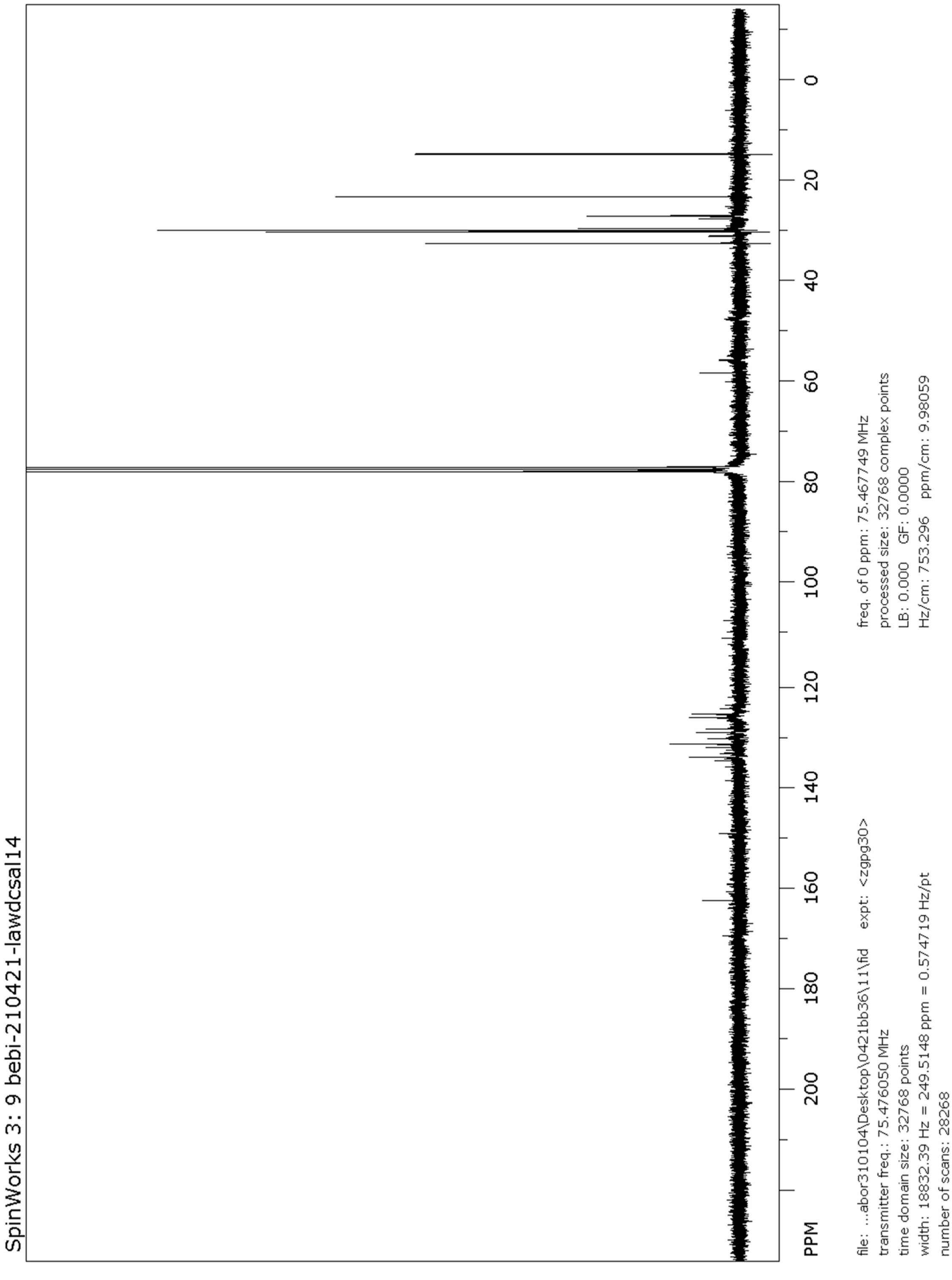

Figure S16. ^{13}C NMR spectrum of **2j**

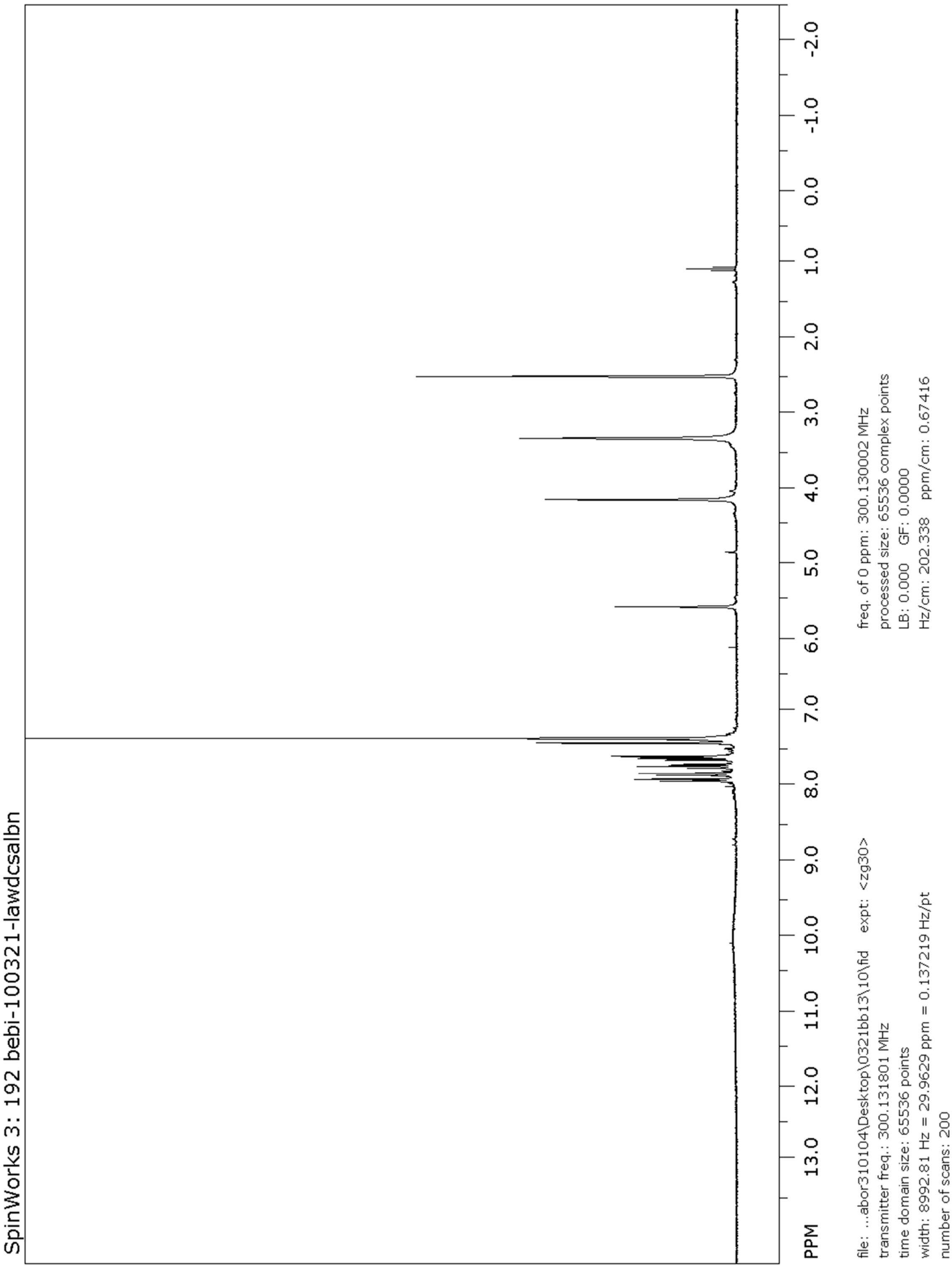

Figure S17. ^{1}H NMR spectrum of **2l**

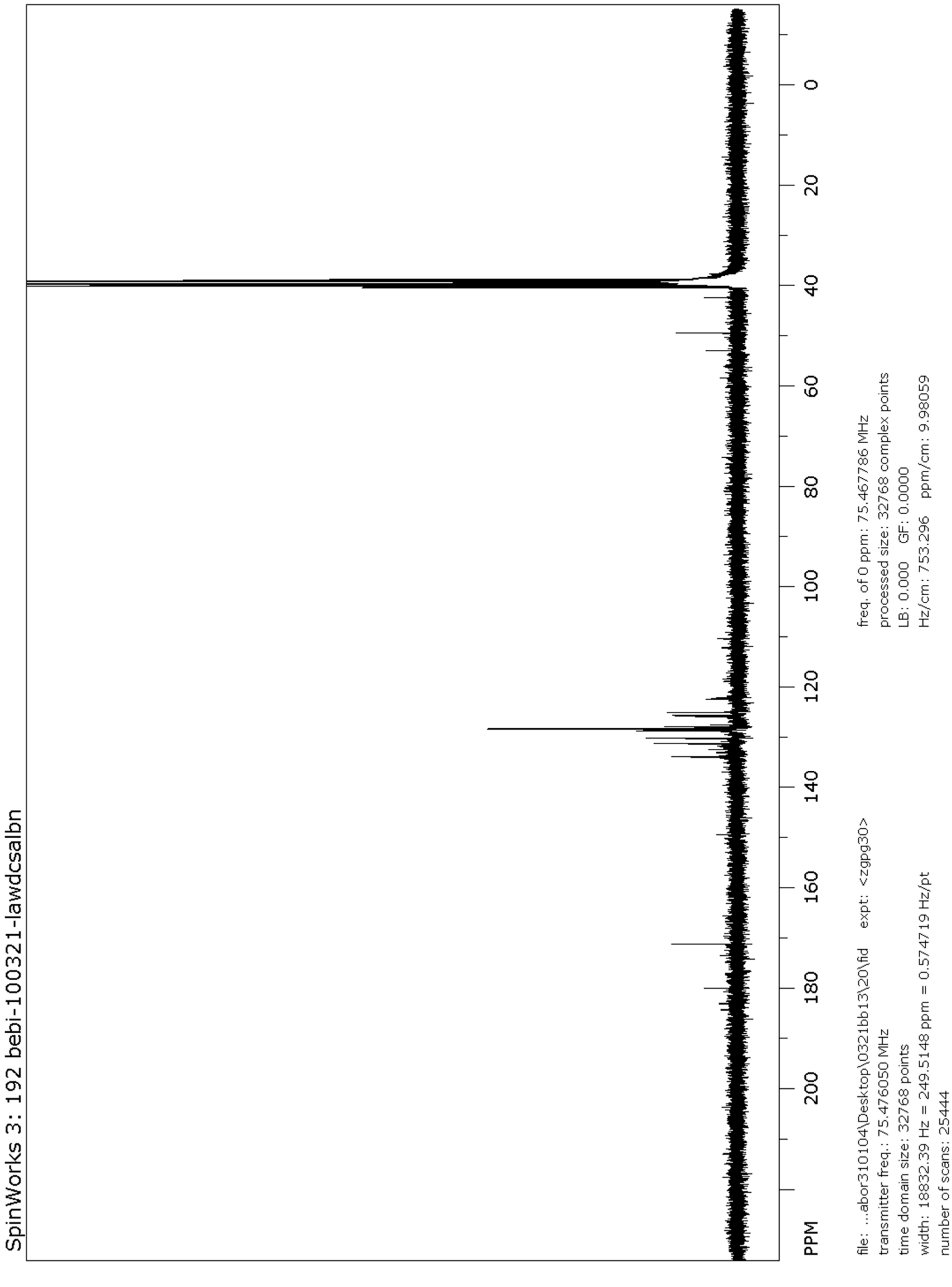

Figure S18. ^{13}C NMR spectrum of **2l**

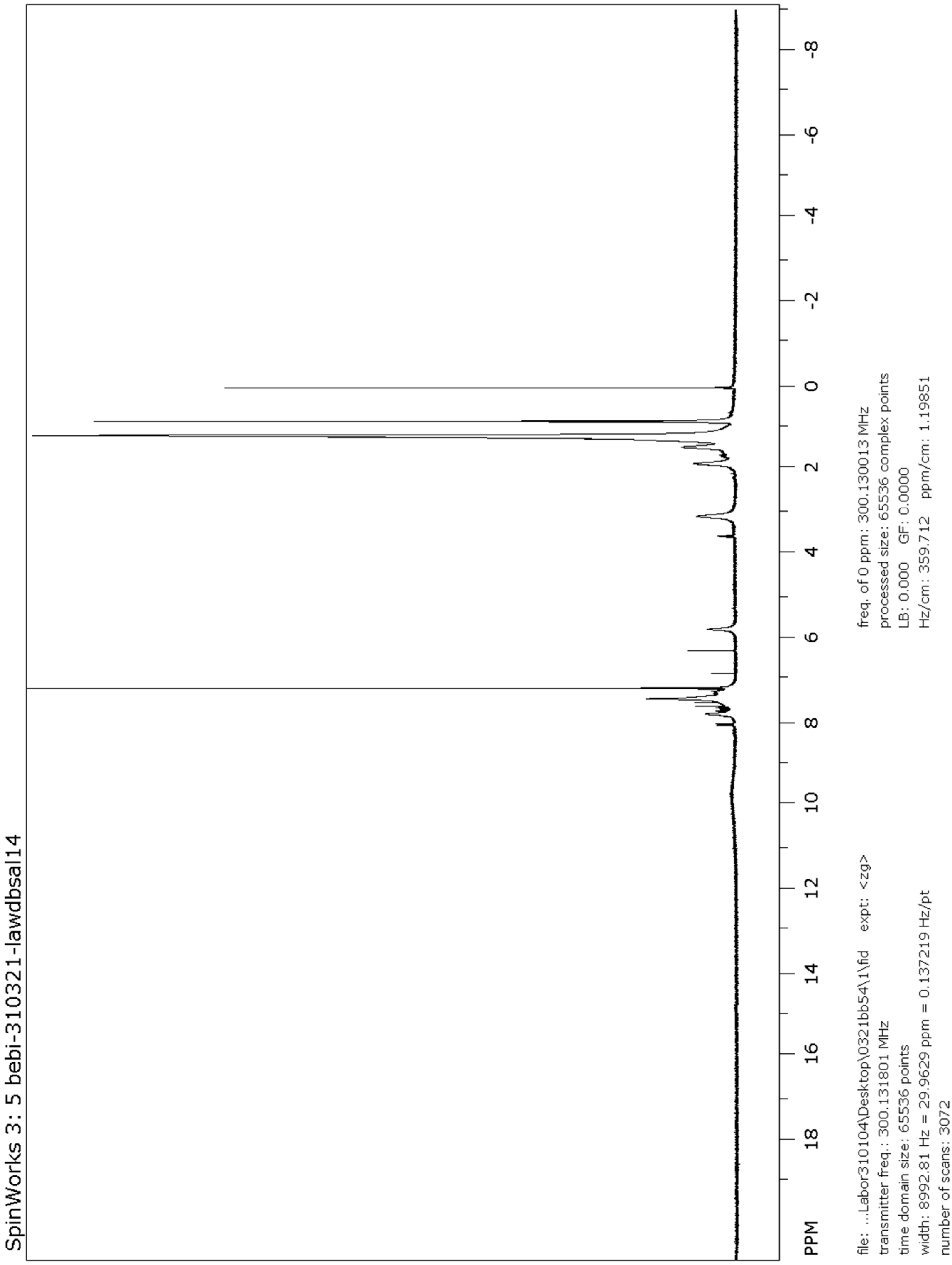

Figure S19. ^{1}H NMR spectrum of **2n**

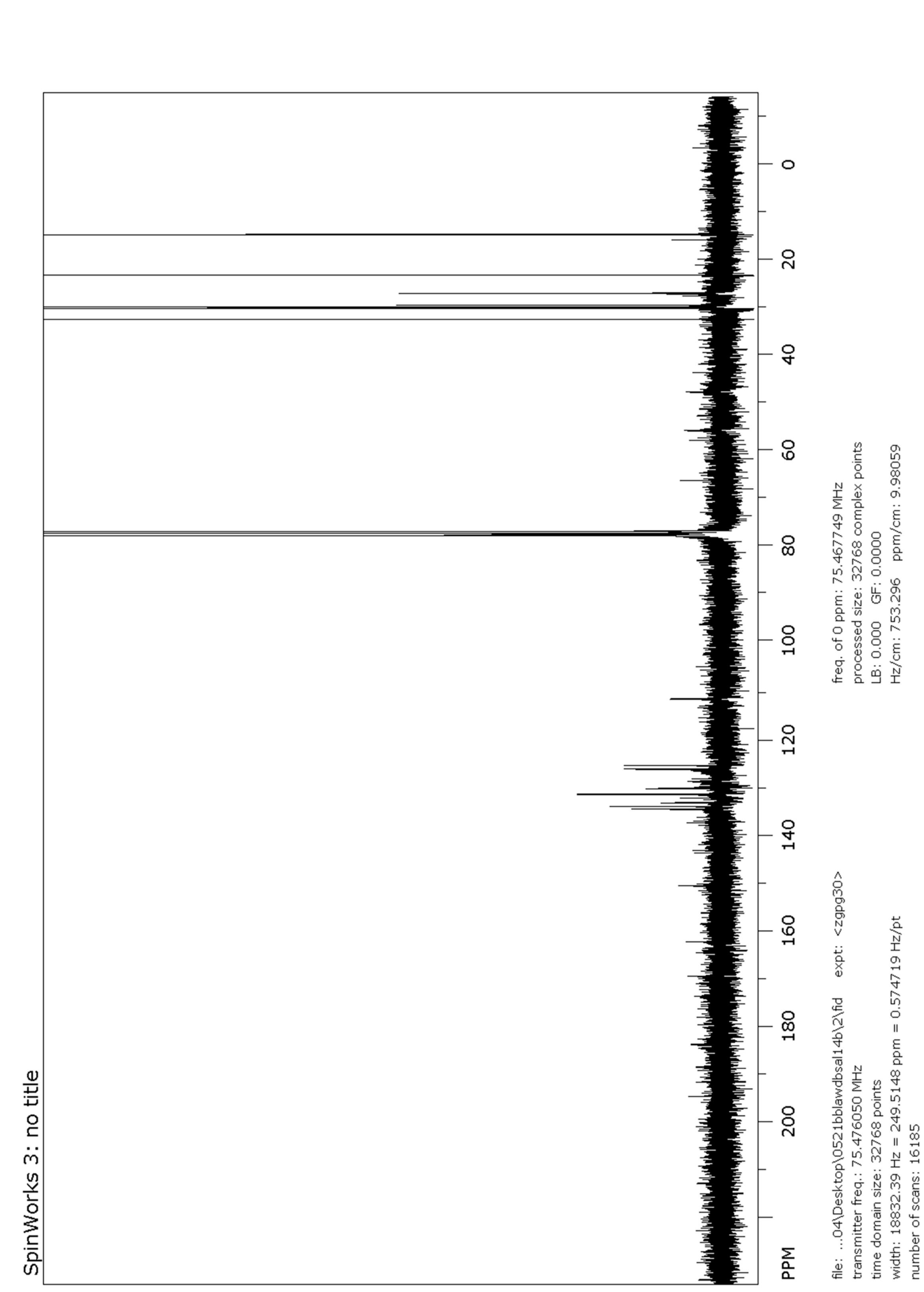

Figure S20. ^{13}C NMR spectrum of **2n**

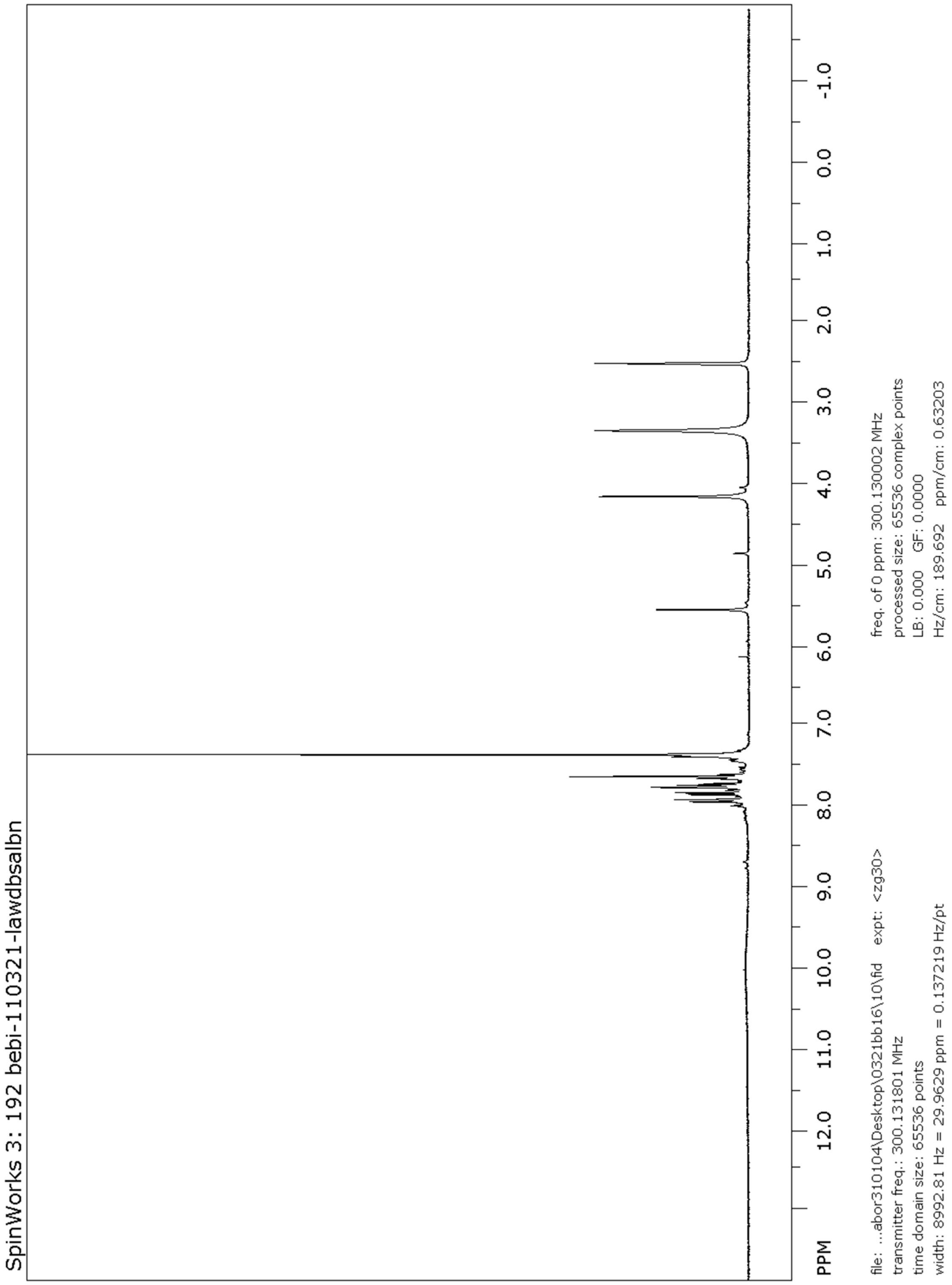

Figure S21. ^{1}H NMR spectrum of **2p**

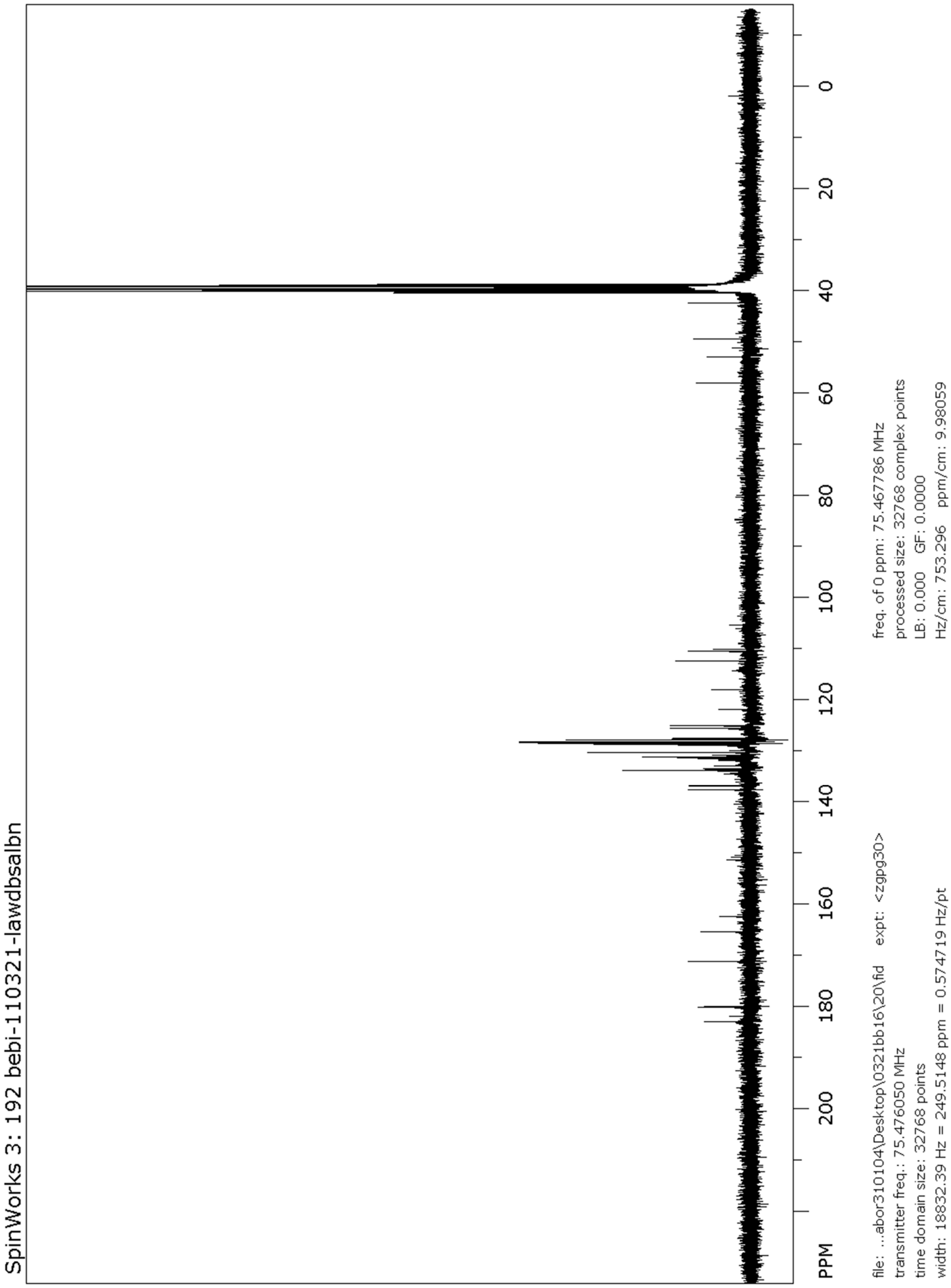

Figure S22. ^{13}C NMR spectrum of **2p**

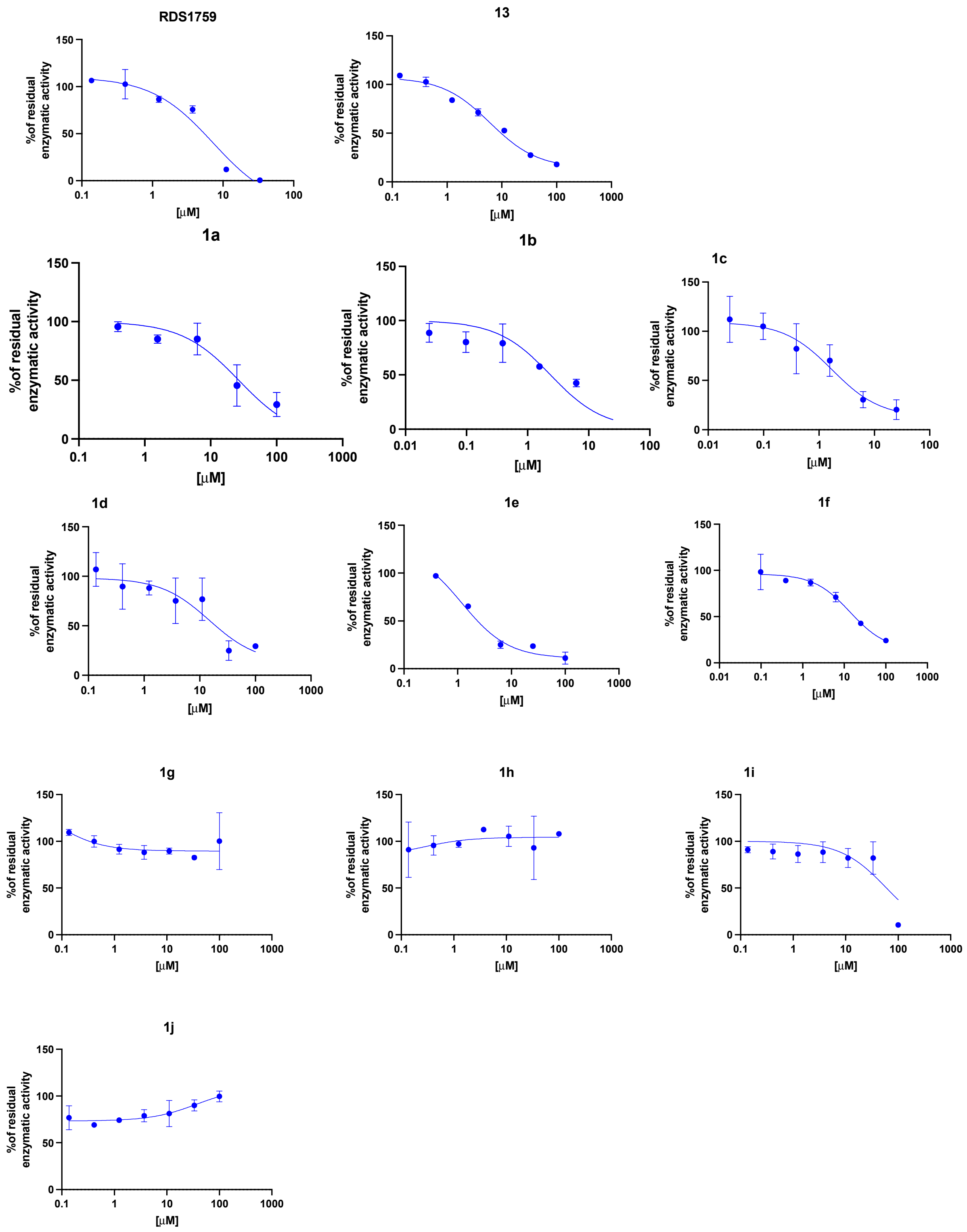

Figure S23. Concentration-dependent inhibition of HIV-1 RT RNase H activity by series **1** compounds

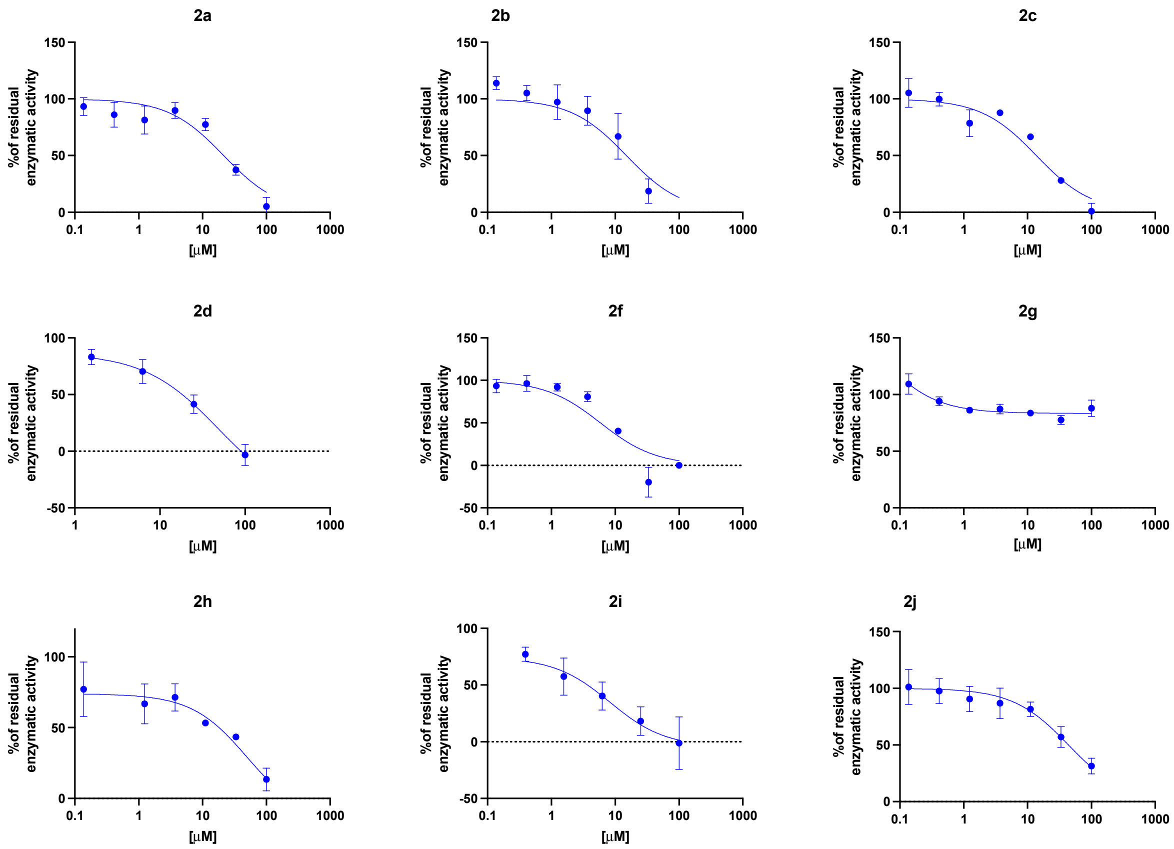

Figure S24. Concentration-dependent inhibition of HIV-1 RT RNase H activity by series **2** compounds

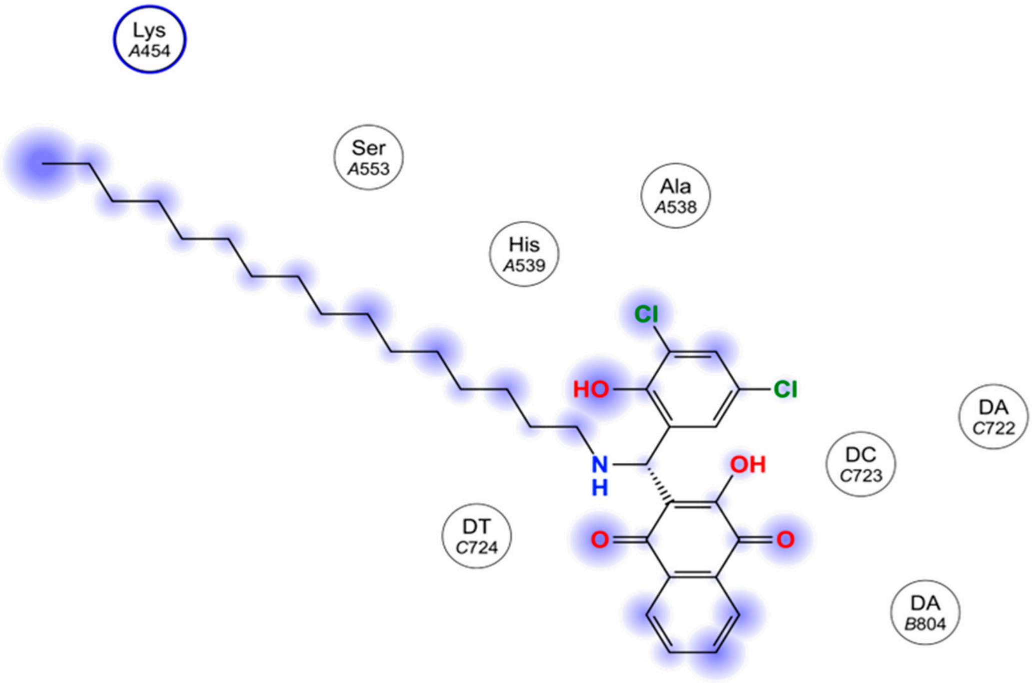

Figure S25: 2D-representation of the docking pose of **2k** with the protein

Details of MD simulations and MM-GBSA methodology:

System Preparation:

1. Protonation States:
   Protonation states of ionizable residues in the protein and DNA were assigned based on their pKa values at physiological pH (7.4) using the *Epik* module integrated within the Schrodinger suite. For the ligand, protonation states of functional groups were similarly calculated to reflect their predominant ionization state at physiological pH.
2. Partial Charges for the Ligand:
   The partial charges for the ligand were derived using the OPLS-2005 force field parameters. Specifically, the *LigPrep* tool was utilized to generate ligand conformations and assign charges optimized for compatibility with the force field.
3. DNA Preparation:
   DNA coordinates were extracted from the crystal structure, and structural integrity was verified using the *Protein Preparation Wizard* in Desmond. Necessary adjustments were made, including the addition of missing hydrogen atoms and assigning proper tautomeric states for the bases. Protonation states of nucleotide residues were assigned based on physiological conditions, ensuring compatibility with simulation parameters.
4. Force Field Considerations:
   While the OPLS-2005 force field was used for the protein and ligand, the DNA was simulated using parameters from the *AMBER ff14SB force field*, which is specifically designed for nucleic acids. This choice ensures accurate representation of the structural dynamics and interactions of DNA within the system. The combination of these force fields was validated within Desmond to ensure compatibility and reliability.
5. Final System Setup:
   The prepared protein-ligand-DNA complex was embedded in a cubic simulation box solvated with SPC water molecules. Sodium ions (Na^+) were added to neutralize the system, and a 0.15 M NaCl solution was included to mimic physiological ionic strength. The system underwent energy minimization and equilibration as described in the methods section to prepare it for production MD simulations.

Energy Minimization:

Energy minimization was performed using the steepest descent algorithm implemented in Desmond 2023-4. The convergence criterion was set to a maximum force threshold of 50 kJ/mol/nm to ensure that the system reached a local energy minimum while resolving steric clashes and eliminating unfavorable geometries.

Equilibration Phases:

The equilibration process was conducted sequentially to ensure gradual stabilization of the system. Initially, the system underwent a 50-nanosecond equilibration phase under an NVT ensemble. This phase aimed to stabilize the temperature of the system at 310 K (37 °C) using the Nosé-Hoover chain thermostat, allowing the system to adapt to thermal conditions while maintaining constant volume. Following this, the system underwent a 12-nanosecond equilibration phase under an NPT ensemble to stabilize pressure at 1 bar using the Martyna-Tuckerman-Klein barostat with a 2 ps relaxation time.

During the equilibration phases, position restraints were applied to heavy atoms of the protein to allow solvent molecules and ions to equilibrate around the solute while maintaining the structural integrity of the protein. The restraint force constant was set to 50 kcal/mol/$Å^2$.

Simulation Parameters:

The van der Waals interactions were modeled with a cutoff distance of 9 Å, which is consistent with the particle mesh Ewald (PME) method used for calculating long-range electrostatic interactions. This parameter was critical for accurately capturing intermolecular forces within the system.

Details for NVT and NPT system:

1. NVT Equilibration Phase (50 Nanoseconds)

Objective: The primary goal of the NVT ensemble equilibration is to stabilize the system by maintaining a constant temperature while allowing the particles to equilibrate in a fixed-volume simulation box. This step ensures that the system does not experience rapid temperature fluctuations, which could lead to non-physical behaviors.

Procedure:

Temperature Control: The system temperature is regulated using a thermostat (e.g., Berendsen thermostat or Langevin dynamics).This thermostat ensures the temperature stabilizes at the desired target, often set to physiological conditions (e.g., 300 K).

Volume Constraints: The simulation box size is kept constant, which is useful for systems where the focus is on stabilizing internal molecular motions without external pressure effects.

Particle Dynamics: Particles are allowed to move and interact under the influence of forces dictated by the potential energy function of the system.

This allows relaxation of steric clashes and initial high-energy configurations.

2. NPT Equilibration and Minimization Phase (12 Nanoseconds)

Objective: The NPT equilibration step introduces constant pressure conditions, allowing the simulation box to adjust its volume dynamically. This phase ensures that the system achieves a realistic density and stabilizes under near-experimental conditions.

Procedure:

Pressure Control:

A barostat (e.g., Parrinello-Rahman barostat or Berendsen barostat) maintains the system at a constant pressure (e.g., 1 atm).Pressure fluctuations allow the simulation box to resize dynamically, adapting to the physical properties of the system.

Density Adjustment: The solvent density is fine-tuned, ensuring that the water molecules (or other solvents) distribute uniformly and occupy realistic volumes around the solute.

Energy Minimization: Residual steric clashes or high-energy configurations are further resolved using energy minimization algorithms (e.g., steepest descent or conjugate gradient methods).

Minimization helps reduce non-physical forces that might have persisted after the NVT phase.

3. Key Differences Between NVT and NPT Equilibration

**Feature**	**NVT Ensemble**	**NPT Ensemble**
**Parameters**	Constant number, volume, temperature	Constant number, pressure, temperature
**Thermodynamic Goal**	Stabilize temperature and particle motion	Stabilize system density and pressure
**Volume**	Fixed	Dynamic (adjustable)
**Applications**	Ideal for temperature equilibration	Ideal for achieving experimental density

Practical Considerations and Applications

Simulation Stability: The two-step equilibration (NVT followed by NPT) ensures that the system is stable and mimics realistic conditions before the production simulation begins.

Protein-Ligand Systems: The ligand-binding pocket adapts during NVT equilibration. In NPT, the pressure stabilization allows the solvent to redistribute optimally around the binding interface.

Membrane Systems: NVT stabilizes the initial configuration of lipids and proteins. NPT allows dynamic adjustments to lipid bilayer thickness, mimicking biological conditions.

Analysis of MD simulations w.r.t RMSD, RMSF, H-bond analysis:

Root Mean Square Deviation (RMSD): RMSD was calculated using the Desmond software by superimposing the trajectory frames onto the reference structure. The fitting was performed on the backbone atoms of the protein to ensure alignment based on structural rigidity. The RMSD values were computed relative to the initial frame of the simulation, representing the unbound structure at time $t = 0$. This approach allowed us to monitor conformational deviations over the course of the trajectory.

Root Mean Square Fluctuation (RMSF): RMSF analysis was conducted to evaluate the flexibility of specific residues over time. Fluctuations were calculated for the alpha carbon ($C\alpha$) atoms of the protein. The analysis was restricted to the equilibrated portion of the trajectory, excluding the initial frames corresponding to the equilibration phase. This ensures that only the stable dynamics of the system are considered in assessing residue-specific flexibility.

Hydrogen Bond (H-bond) Analysis: Hydrogen bonds were identified and analyzed using Desmond's default parameters, which define an H-bond as a donor-acceptor pair with a maximum distance of 2.5 Å and a donor-hydrogen-acceptor angle greater than 120°. These stringent criteria ensure that only strong, well-defined hydrogen bonds are included in the analysis.

Details of Molecular Docking:

Step 1: Protein Preparation

Proper preparation of the protein structure is essential for accurate docking results. This step ensures that the protein structure is free of errors and ready for docking.

1.1 Load the Protein

Download the structure of the protein in PDB format from the Protein Data Bank (PDB).

Open the structure using AutoDockTools (ADT), which is the graphical interface for AutoDock.

1.2 Adding Hydrogens

In ADT, select the option to Add Hydrogens.

Add polar hydrogens (i.e., hydrogens on electronegative atoms like oxygen and nitrogen). This ensures the proper protonation state of acidic (e.g., Glu, Asp) and basic residues (e.g., Lys, Arg).

1.3 Assignment of Partial Charges

Kollman Charges: AutoDock uses Kollman charges to calculate electrostatic interactions.

In ADT, navigate to the Edit > Charges > Add Kollman Charges menu.

The Kollman charge assignment will automatically distribute partial charges across the protein atoms based on standard parameters for each atom type.

Step 2: Ligand Preparation

The ligand preparation ensures that the small molecule is compatible with AutoDock and can interact with the protein.

2.1 Load the Ligand

Open the ligand structure file in PDB or MOL2 format using ADT.

2.2 Add Hydrogens and Charges

Similar to the protein, add polar hydrogens to the ligand.

Use AutoDockTools to convert the ligand structure to PDBQT format, which includes torsional degrees of freedom.

Step 3: Saving the Protein and Ligand

Save the prepared protein structure as a PDBQT file.

Save the ligand as a PDBQT or Mol2 (use PyMOL for this conversion) file after

assigning charges and torsions.

4.2 Run Docking

Use Genetic Algorithm (GA) as the docking algorithm in NRGSuite. Follow the procedure given in the manual of the software for optimum results.

Execute the docking run and analyze the docking poses and binding affinities.

Tools Used

AutoDockTools: For adding hydrogens, assigning Kollman charges, and converting PDB to PDBQT format.

NRGSuite: For actual docking.

Molecular Visualization: Tools like PyMOL for visualizing adjustments.
